# Real-Time Segmentation and Classification of Birdsong Syllables for Learning Experiments

**DOI:** 10.1523/ENEURO.0023-26.2026

**Published:** 2026-06-30

**Authors:** Nils Riekers, Jacqueline Laura Göbl, Franziska Heubach, Lena Veit

**Affiliations:** Neurobiology of Vocal Communication, Institute for Neurobiology, University of Tübingen, Tübingen 72076, Germany

**Keywords:** annotation, closed-loop, labeling, vocal sequence, vocalization, voice activity detection

## Abstract

Songbirds are essential for studying neuronal mechanisms of learned vocalizations. Closed-loop interventions require online recognition of a specific target syllable while the bird is singing, for example, for manipulation of auditory feedback, song-triggered neuronal microstimulation or optogenetics. Existing tools for closed-loop interventions can recognize only single syllables through manually created templates, with limited flexibility to adapt to new experiments. We here present Moove (Marking Online using only the Onsets of Vocal Elements), a novel neural network approach to real-time syllable segmentation and classification of Bengalese finch songs. Moove's two-stage architecture detects syllable onsets and offsets and classifies syllables using acoustic information only from the first part of the syllable, enabling precise temporal contingency between behavior and feedback. We verify Moove's fast and accurate online annotation of all recorded syllables in five adult male Bengalese finches (*Lonchura striata domestica*). To validate Moove as a tool for learning experiments, we trained one adult male Bengalese finch with an established protocol: a specific target syllable is covered with noise, which masks auditory feedback and leads the bird to introduce specific modifications to syllable sequencing. The trained bird learned to avoid the targeted syllable sequence with comparable outcomes to previous reinforcement learning experiments. Our results show that Moove can correctly segment and classify Bengalese finch syllables in real time, with speed and reliability that allows effective operant conditioning experiments. Moove could be used for other closed-loop experiments on vocal signals, making it a crucial tool for future investigations of birdsong sequencing.

## Significance Statement

Moove (Marking Online using only the Onsets of Vocal Elements) is a new tool for the real-time annotation of birdsong. It annotates song syllables while they are still being sung, allowing experimental manipulations such as reinforcement, auditory feedback disruption, or stimulation to be applied to ongoing syllables. Moove uses a two-stage architecture with convolutional neural networks for segmentation and classification. We verify Moove's functionality in a reinforcement learning experiment by training a Bengalese finch to change its song sequence through disturbed auditory feedback. Moove is easy to use with a graphical user interface for network training and manual verification. Its open-source Python code can be adapted to a wide range of applications on vocal signals.

## Introduction

Songbirds are ideally suited to study learned vocal communication (Doupe and Kuhl, 1999; Brainard and Doupe, 2013). In many species, song is composed of acoustically discrete vocal elements, called syllables. Bengalese finches flexibly arrange these syllables into variable sequences following learned syntactic rules (Okanoya and Yamaguchi, 1997; Okanoya, 2004; Veit et al., 2021; Koparkar et al., 2024). We here use letters (e.g., “a,” “b,” “c”) to label distinct syllable types.

Immense progress has been made deciphering the neuronal mechanisms of song production and learning by interfering with song in closed-loop experiments (Tumer and Brainard, 2007; Andalman and Fee, 2009; Sober and Brainard, 2012). Important approaches like song-triggered manipulations of auditory feedback (Sakata and Brainard, 2006; Tumer and Brainard, 2007; Sober and Brainard, 2009; Gadagkar et al., 2016; Riedner and Adam, 2020), visual feedback (Cynx, 1990; Franz and Goller, 2002; Seki et al., 2008; Zai et al., 2020) or somatosensory feedback (McGregor et al., 2022), song-triggered optogenetic stimulation (Roberts et al., 2012; Hisey et al., 2018; Xiao et al., 2018), microstimulation (Ashmore et al., 2005; Kao et al., 2005; Moll et al., 2023), or other behavioral contingencies (Carouso-Peck and Goldstein, 2019; Kawaji et al., 2024) require the online recognition of target syllables and immediate delivery of feedback while the bird is singing. One key protocol introduces learned changes to the song using reinforcement learning. By masking a specific target syllable with a short burst of aversive white noise (WN), birds learn to modify their song to avoid triggering the WN in future renditions. This protocol has been used to modify syllable pitch (Tumer and Brainard, 2007; Andalman and Fee, 2009; Warren et al., 2012; Canopoli et al., 2014; Tian and Brainard, 2017; Tian et al., 2023; Zai et al., 2024), timing (Ali et al., 2013; Tachibana et al., 2017), and song sequencing in Bengalese finches (Warren et al., 2012; Veit et al., 2021; Kawaji et al., 2024; Fortkord and Veit, 2025).

Current technical solutions for closed-loop experiments range from manual triggering (Carouso-Peck and Goldstein, 2019; Phaniraj et al., 2022) to spectral template-matching (Kao et al., 2005; Tumer and Brainard, 2007; Fortkord and Veit, 2025) and neural networks (Steinfath et al., 2021; Kawaji et al., 2024). Template-based solutions achieve low latencies but require manually designed templates for target syllables, which need to be manually adapted to syllable changes introduced by the bird. The creation of an optimal template is challenging and substantially influences the accuracy of syllable recognition (Kulkarni and Troyer, 2020). Recognizing syllables in specific sequences is possible by combining multiple templates with Boolean logic and latencies between template matches, but this quickly becomes unwieldy for species with variable sequencing.

Several neural network approaches have been developed for closed-loop targeting of birdsong (Pearre et al., 2017), offline annotation (Cohen et al., 2022), or both (Steinfath et al., 2021). Frame classification models (Graves, 2012) such as DAS (Steinfath et al., 2021) and TweetyNet (Cohen et al., 2022) assign a label to each audio frame and recover syllable segments through postprocessing. Another approach is to first detect the onsets and offsets of syllable segments (“segmentation”) and then classify sound segments into syllable types (Schulthess et al., 2023; Kawaji et al., 2024). Existing tools that follow this two-stage process classify syllable identity only after the segment's offset. However, in many experiments, it is essential to target specific syllable sequences while they are still being vocalized (Tumer and Brainard, 2007; Charlesworth et al., 2011).

**Figure 1.**
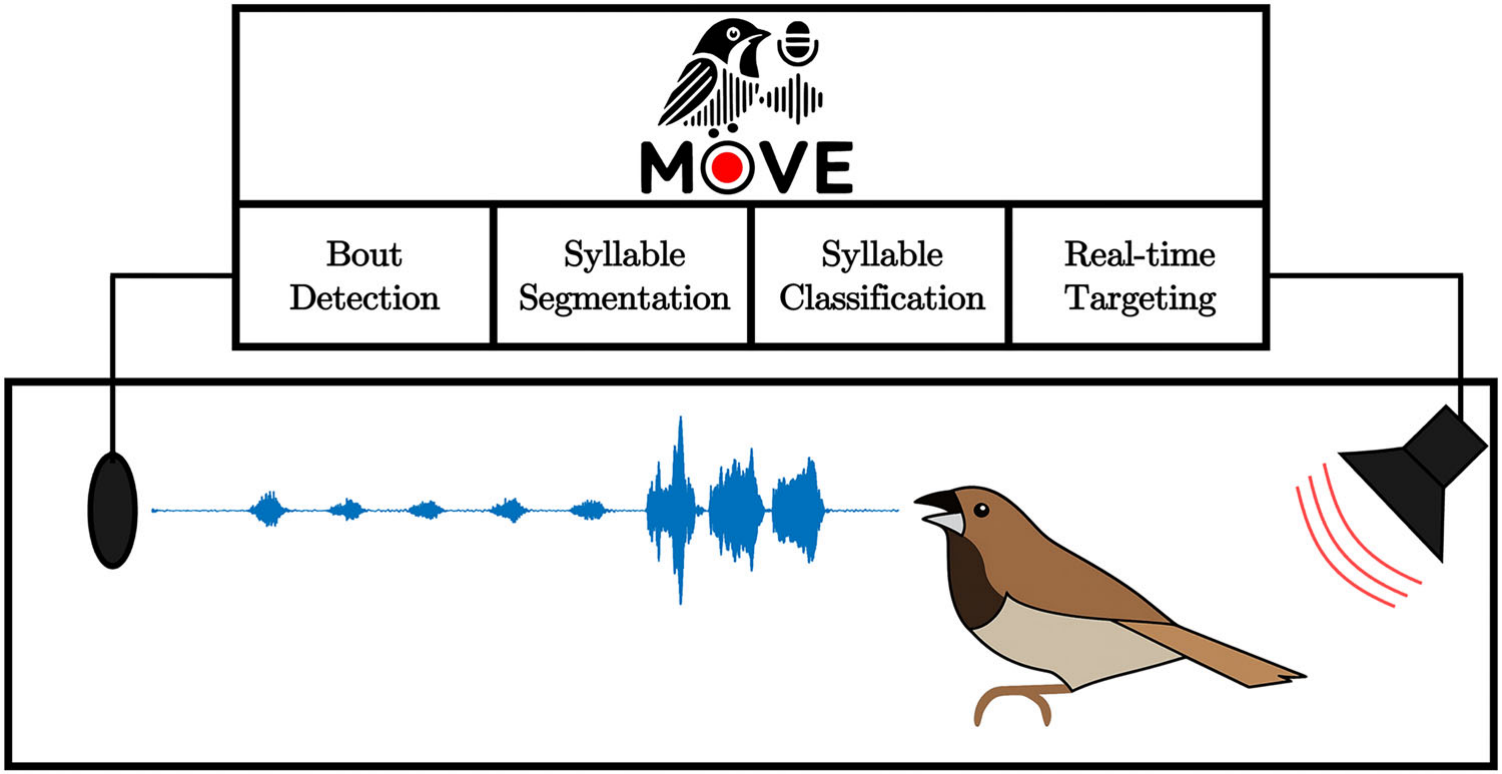
Functional overview of Moove. The system continuously monitors vocalizations from a singing Bengalese finch and performs four sequential processing steps: (1) Bout detection identifies song bouts from continuous audio input, (2) syllable segmentation detects onsets and offsets of individual syllables within detected bouts, (3) syllable classification assigns syllable types using only the initial portion of each syllable, and (4) real-time targeting triggers feedback stimuli (e.g., WN playback), while the target syllable is still being produced. The diagram illustrates the complete closed-loop system from audio acquisition (microphone, left) through neural network processing (Moove pipeline, top) to feedback delivery (speaker, right), enabling operant conditioning experiments with short latencies.

This motivated us to develop Moove (Marking Online using only the Onsets of Vocal Elements). Similar to Schulthess et al., Moove uses a lightweight binary classifier for rapid onset detection, followed by a separate classification network. Moove classifies syllables using acoustic information from the beginning of each syllable, rather than waiting for offset, which enables feedback delivery while the syllable is still being sung. This open-source tool for real-time birdsong annotation is easy to modify and works without specialized hardware (Fig. 1).

## Materials and Methods

Moove is a Python tool for real-time birdsong syllable segmentation and classification using neural networks. The package consists of MooveTAF (Moove Targeted Auditory Feedback) for song recording and real-time targeting, and MooveGUI for data preprocessing, manual labeling, and network training.

### Code accessibility

The code/software described in the paper is freely available online at https://github.com/veitlab/moove and is available as Extended Data 1. The code is implemented in Python and was primarily developed and tested on a standard desktop PC with an Intel Core i5 CPU running Windows 11. It is compatible with common operating systems and off-the-shelf audio interfaces, and no specialized hardware is required.

10.1523/ENEURO.0023-26.2026.d1 Data 1 The complete Python source code for MooveTAF (real-time recording and targeting) and MooveGUI (data preprocessing, labeling, and network training), packaged as a single ZIP archive. The latest version is available at: https://github.com/veitlab/moove Download Data 1, ZIP file.

### MooveTAF: recording and real-time targeting

MooveTAF was developed to provide audio recording and real-time targeting of birdsong syllables, enabling online segmentation, classification, and feedback triggering. It uses the Python package *sounddevice* (Geier, 2020) as an interface to PortAudio (Bencina and Burk, 2001) and supports multiple native audio APIs. The name and several of its features are inspired by the LabVIEW program EvTAF (Tumer and Brainard, 2007). An important goal was to replace and extend the functionalities of EvTAF while at the same time ensuring compatibility through similar file types and annotation formats to facilitate adoption by research groups which are already using this software (including our own).

To enable real-time segmentation and classification of song syllables, a key design requirement was to balance high accuracy with fast inference time. The inference speed of the system must be faster than the rate at which new audio chunks, small consecutive sections of the audio stream, arrive, to prevent delays from accumulating and negatively impacting the tool's real-time capability. The size of audio chunks is therefore one of the major contributors to latency in real-time audio classification (Steinfath et al., 2021). Smaller chunks reduce latency and allow for faster inference but limit the temporal context available for classification. To address this, we implemented separate dedicated networks for segmentation and classification with different chunk sizes, thereby reducing latency.

### Architecture of MooveTAF

The segmentation network is a dedicated network for the detection of syllable segments in a continuous stream of audio data. It operates on very short chunks to perform binary frame-level classification of the presence or absence of syllables. A second network (the classification network) classifies the detected syllable segments into syllable types. After the segmentation network has detected a syllable onset, the following audio data are collected over a short period of time (default, 30 ms) and then transferred to the classification network. This approach of first detecting syllable segments and then classifying them into types is similar to the architectures of Schulthess et al. (2023) and Kawaji et al. (2024).

**Figure 2.**
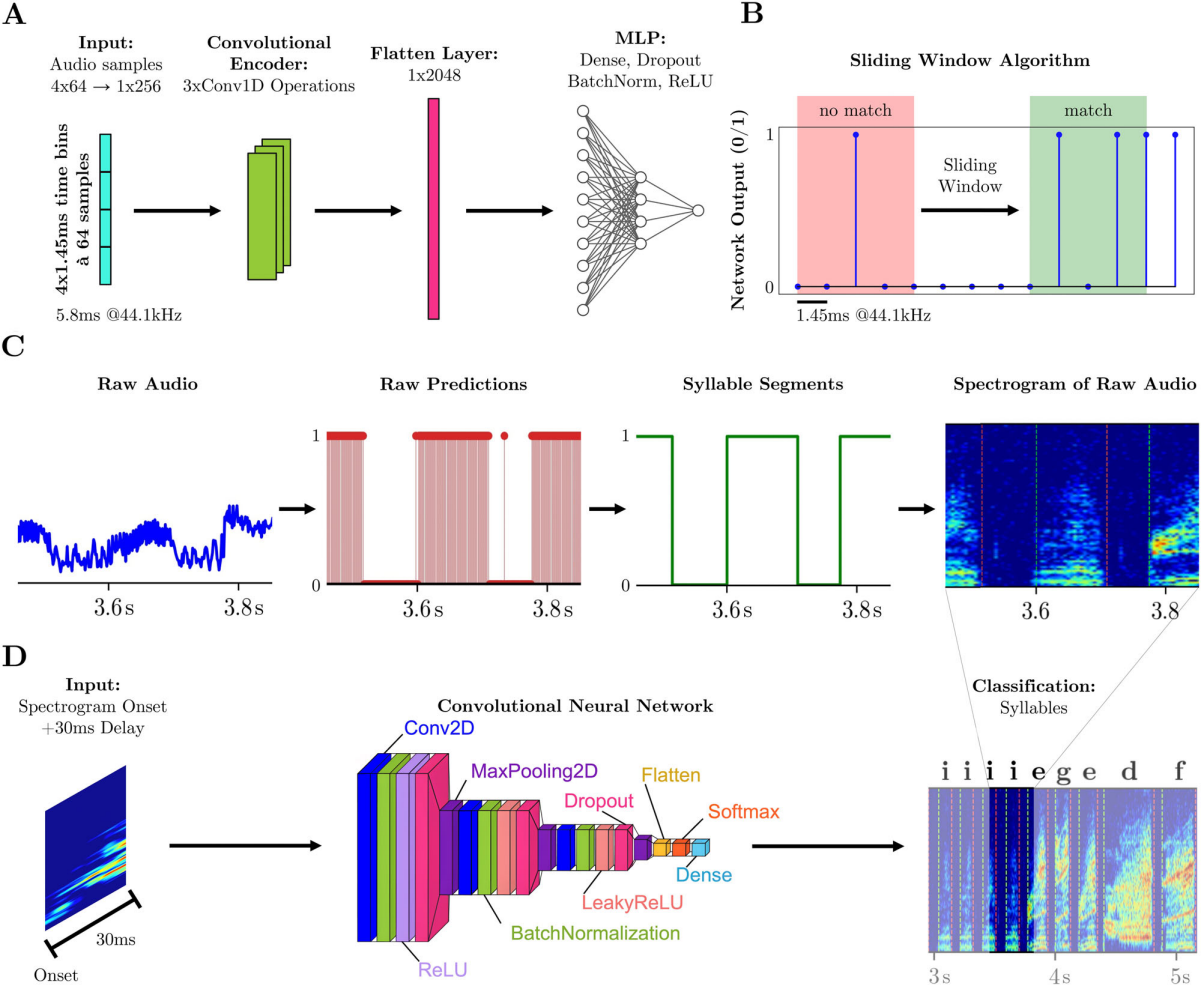
Architecture of the two-stage neural network system in MooveTAF. ***A***, Segmentation network architecture. The network receives consecutive audio chunks (64 samples at 44.1 kHz) together with the three preceding chunks as input. A convolutional encoder extracts features from the raw audio, followed by a MLP that predicts whether the current chunk belongs to a syllable segment (output, 0 or 1). ***B***, Sliding window algorithm for onset detection. A syllable onset is confirmed when at least three out of five consecutive chunks in a sliding window are classified as belonging to a syllable segment (green). When fewer than three chunks are classified as syllable (red), no onset is detected. ***C***, Example of segmentation output showing the complete processing pipeline. From left to right, Raw audio waveform, raw binary predictions from the segmentation network (red bars with white vertical lines indicating individual chunk predictions), merged syllable segments after applying the sliding window algorithm (green boxes), and spectrogram of the raw audio with vertical dashed lines marking the detected syllable boundaries. ***D***, Classification network architecture. After syllable onset detection, 30 ms of audio are buffered and converted to a spectrogram. This spectrogram is processed by a convolutional neural network (CNN) consisting of three convolutional blocks (Conv2D, BatchNormalization, LeakyReLU/ReLU, Dropout, MaxPooling2D), followed by a flatten layer, a fully connected layer, and softmax activation to assign a syllable label (e.g., “a,” “b,” “c”).

The segmentation network (Fig. 2*A*) determines whether each incoming audio chunk is part of a syllable segment. It consists of a convolutional encoder to extract features and a multilayer perceptron (MLP) that classifies each frame as belonging to either a syllable class or the background class, consisting of silent gaps and all other nonsyllable sounds. Using the default parameters of a chunk size of 64 samples at 44.1 kHz, this network makes ∼690 inferences per second. Due to this high number of inferences, errors inevitably occur even at high accuracy levels. Therefore, a sliding window algorithm (Fig. 2*B*) was implemented which identifies syllable onsets when a predefined number of chunks within a sliding window are classified as part of a syllable segment (default: 3 out of 5 consecutive chunks). This postprocessing step reduces false positives from individual chunk predictions and yields continuous syllable segments that correspond well to syllables in the spectrogram (Fig. 2*C*).

Once a syllable onset is detected, audio data are buffered for a short period (default: 30 ms; ∼30.47 ms at 44.1 kHz, chunk size 64) and transformed into a spectrogram, which serves as input for the classification network (Fig. 2*D*). The classification network, a CNN, then assigns a label to the syllable segment based on this input. If the classified syllable sequence matches a user-defined target sequence, MooveTAF can trigger a feedback stimulus (e.g., WN playback), enabling real-time closed-loop experiments.

Syllable sequences in Bengalese finch song contain different sequence structures such as chunks, where syllables follow a fixed order (such as “a–b–c”), branch points, where multiple different syllables may follow the same syllable type, as well as repeat phrases, where the same syllable type is repeated a variable number of times (Seki et al., 2008; Suge and Okanoya, 2010; Koparkar et al., 2024). For real-time targeting, one or several target sequences can be defined by the user. If multiple target sequences are defined, one is randomly selected at the start of each bout. Once targeting is enabled, MooveTAF continuously monitors the detected syllable stream and applies regular expression-based pattern matching to determine whether the detected syllable sequence matches the currently active target sequence. Regular expression pattern matching accommodates both naturally occurring song sequence variability and occasional classification errors. For example, the pattern [jc]a$ triggers feedback for sequences ending in either “j–a” or “c–a.” Regular expression targeting rules can include or exclude specific sequence contexts or specify a range of repeat counts for repeat phrases. This enables precise experimental control over which vocal sequences trigger feedback.

If the currently classified syllable sequence matches the currently active target pattern, a feedback stimulus is triggered. A catch-trial rate can be set to omit triggering in a defined proportion of bouts. By default, the feedback stimulus consists of playback of a user-provided wav file (e.g., WN), though the system can be readily adapted for other feedback modalities. All parameters of the targeting behavior, such as the target sequence, feedback stimulus file, and catch-trial rate, are user-definable in the config file. If multiple feedback stimulus files are provided, MooveTAF randomly selects one with replacement for every detected target.

### MooveGUI: data preprocessing, labeling, and network training

**Figure 3.**
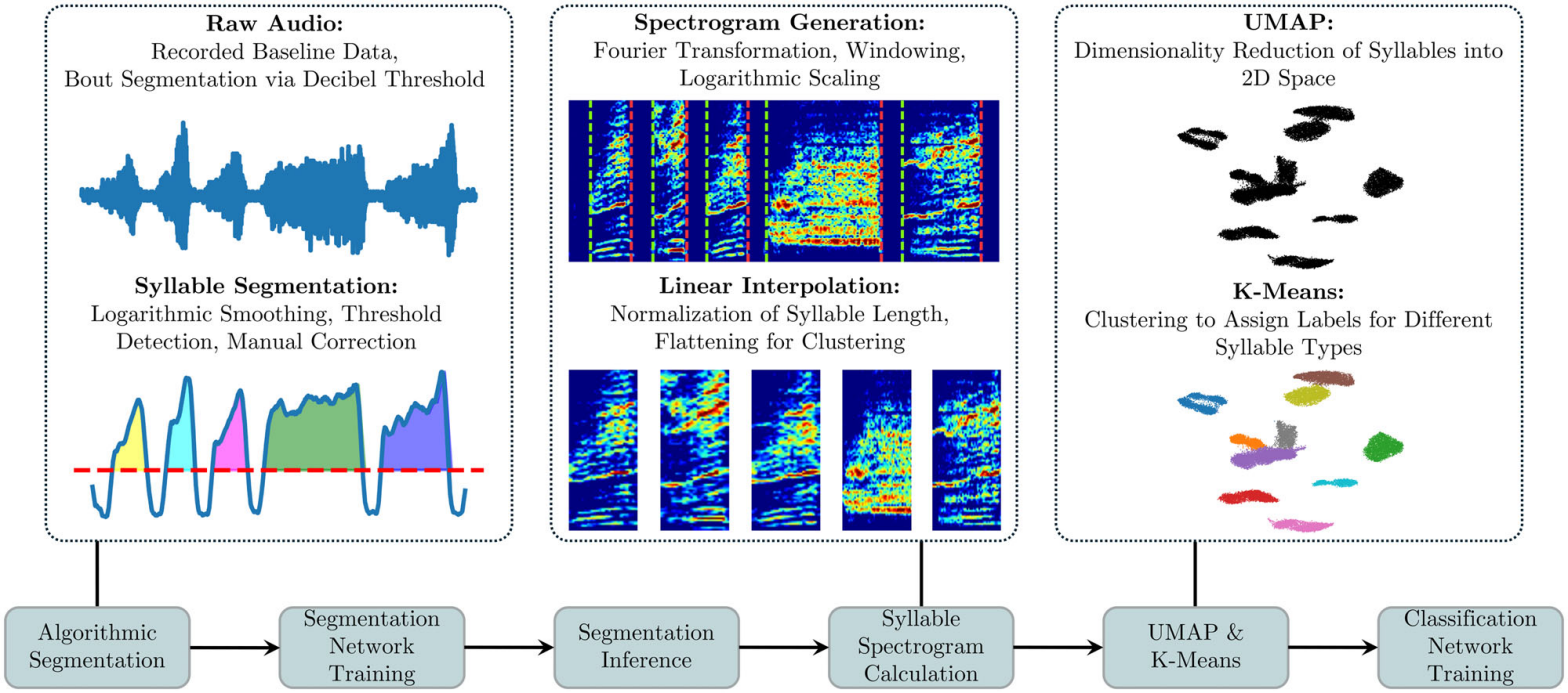
Data preprocessing and network training pipeline in MooveGUI. Left panel, Syllables are first segmented using an energy-based threshold method and, if necessary, manually corrected. The verified segments are then used to train the segmentation network. Subsequently, the trained network is applied to resegment the data, ensuring consistency with the segmentation that will be performed during real-time experiments. Middle panel, From the resulting syllable segments, spectrograms are computed using a STFT, normalized by linear interpolation to unify syllable length, and flattened into feature vectors. Right panel, these vectors are projected into a two-dimensional space using UMAP to visualize acoustic similarity between syllables, and *k*-means clustering is applied to obtain initial syllable labels (colored clusters). Cluster assignments can then be manually refined via the GUI in the UMAP space or in the spectrogram to correct mislabels or merge entire clusters. Finally, the classification network is trained on spectrogram inputs paired with the curated labels, using the same temporal window (e.g., 30 ms) as during later online inference.

Before syllable targeting can be performed, baseline recordings must be collected using MooveTAF in recording mode (segmentation, classification, and feedback disabled). Bouts are detected and saved via an energy-based (sound amplitude) method using criteria such as minimum bout length. As illustrated in Figure 3, all subsequent data preprocessing and training steps are conducted in MooveGUI, which is fully integrated into the Moove Python package. MooveGUI provides a user-friendly, PyQt6-based graphical interface that allows users to execute all necessary preprocessing steps interactively and to train both neural networks directly within the tool. The segmentation and classification networks are trained separately for each bird, based on its individual song repertoire. Once training is complete, the resulting models can be loaded into MooveTAF to perform real-time segmentation, classification, and targeting during closed-loop experiments. This semiautomated pipeline thus provides a cohesive workflow from data collection to model deployment. The following sections describe each step of this preprocessing and training pipeline in detail.

#### Energy-based segmentation

In the first step, each bout of raw audio data is segmented using an energy-based method based on the algorithm implemented in *evfuncs* (Nicholson, 2021), which applies bandpass filtering, amplitude squaring, smoothing, and conversion into decibel values. An amplitude threshold is then used to detect syllable onsets and offsets, while segments shorter than a minimum duration are excluded, and segments separated by silent intervals shorter than a minimum duration are merged. This functionality has been integrated into MooveGUI, which also allows manual refinement of onset and offset times. Since energy-based segmentation is prone to over- and undersegmentation (Cohen et al., 2022), manual corrections are often required to obtain consistent segmentation and thus accurate training data for the segmentation network.

#### Segmentation network training

Once a sufficient number of training bouts have been segmented, the segmentation network can be trained. The training can proceed iteratively: the manually segmented bouts are used to train an initial model, which is then refined using bouts segmented by the model, which are manually corrected and added to the training data. This process allows large numbers of training bouts to be obtained relatively quickly, gradually improving the model with each iteration (Steinfath et al., 2021).

Segmented bouts are randomly divided into training (70%), validation (15%), and test (15%) subsets. The segmentation network is trained using binary cross-entropy loss with positive class weighting to address class imbalance between syllable and background frames, the Adam optimizer (learning rate, 0.001; batch size, 64), and early stopping with a patience of five epochs to prevent overfitting when validation loss no longer improves.

#### Segmentation inference

Once the segmentation network is trained, it is applied to resegment the training data using the same sliding window algorithm that will be used during real-time operation. This resegmentation ensures consistency between offline training data and online syllable detection.

#### Syllable spectrogram calculation

For each syllable segment, spectrograms are computed using a short-time Fourier transform (STFT). The same STFT parameters (window length, overlap, FFT size, and frequency cutoffs) are applied to all syllables. Syllable durations are normalized by linear interpolation to obtain equal-size spectrograms (Koparkar et al., 2024), and the amplitude of each spectrogram is converted into decibel values. The resulting spectrograms serve as input for subsequent dimensionality reduction and clustering.

#### UMAP and *K*-means

The spectrograms of detected syllable segments are projected into a two-dimensional space using the Uniform Manifold Approximation and Projection (UMAP; McInnes et al., 2018) algorithm (Sainburg et al., 2020; Steinfath et al., 2021; Kawaji et al., 2024; Koparkar et al., 2024), where syllables with similar spectral structure cluster together, resulting in distinct clusters for different syllable types in Bengalese finch song. The *k*-means algorithm (MacQueen, 1967) is then applied to these embeddings to assign each syllable segment to one of the clusters and thus apply labels. UMAP parameters (number of neighbors and minimum distance) and number of clusters for *k*-means can be adjusted by the user.

#### Manual adjustments and verification of cluster membership (GUI-based)

MooveGUI includes an interface for manual adjustment of cluster memberships, where users can visually inspect the assigned labels in the 2D UMAP projection and directly reassign data points. This allows manual splitting or merging of clusters or correction of mislabels.

#### Classification network training

The classification network is trained on spectrograms of syllable segments together with their manually verified labels. To enable low-latency online classification, only a fixed temporal window after each detected onset (default, 30 ms) is used as input during both training and inference. The network is optimized using cross-entropy loss, the Adam optimizer (learning rate, 0.001; batch size, 64), and early stopping with a patience of five epochs. To improve generalization, data augmentation was applied to training spectrograms with a probability of 20% per sample. For each augmented sample, one of four augmentation techniques is randomly selected: additive Gaussian noise (noise level, 0.0001), frequency masking (up to 10 frequency bins zeroed), time masking (up to 10 time frames zeroed), or dynamic range compression (logarithmic compression with factor 0.5). No augmentation was applied to the segmentation network. Dataset splits and normalization are performed as described for the segmentation network training. Class imbalance is addressed through inverse-frequency class weighting. After training the classification network, Moove can be used to reliably distinguish syllable types online during real-time experiments.

### Animals

Five male Bengalese finches were used to test different features of Moove across multiple experiments. Birds were housed individually in sound-attenuating chambers (120 × 50 × 50 cm) under a 14:10 h light–dark cycle (lights on 07:00–21:00; ZT0 = 07:00) at 25°C and 50% humidity, with *ad libitum* access to food, water, and standard enrichment (sand baths, nests, fresh greens). The light–dark transitions included a 30 min gradual dimming period (06:30–07:00 and 20:30–21:00). Individual housing was required for the duration of recordings for isolated acoustic recordings. Recordings were performed during the light phase. Between experimental sessions, birds were returned to large group aviaries. Vocalizations were recorded using a Rode M5 MP microphone (Australia) connected to a Steinberg UR12/IXO12 audio interface (Germany), with a JBL Control 1 Pro loudspeaker (USA) used for playback. All recordings were made using Moove's default configuration settings as specified in the documentation. All experimental procedures were approved by the Regierungspräsidium Tübingen (Germany) and conducted in compliance with animal welfare regulations.

### Experiment 1: system performance characterization

**Figure 4.**
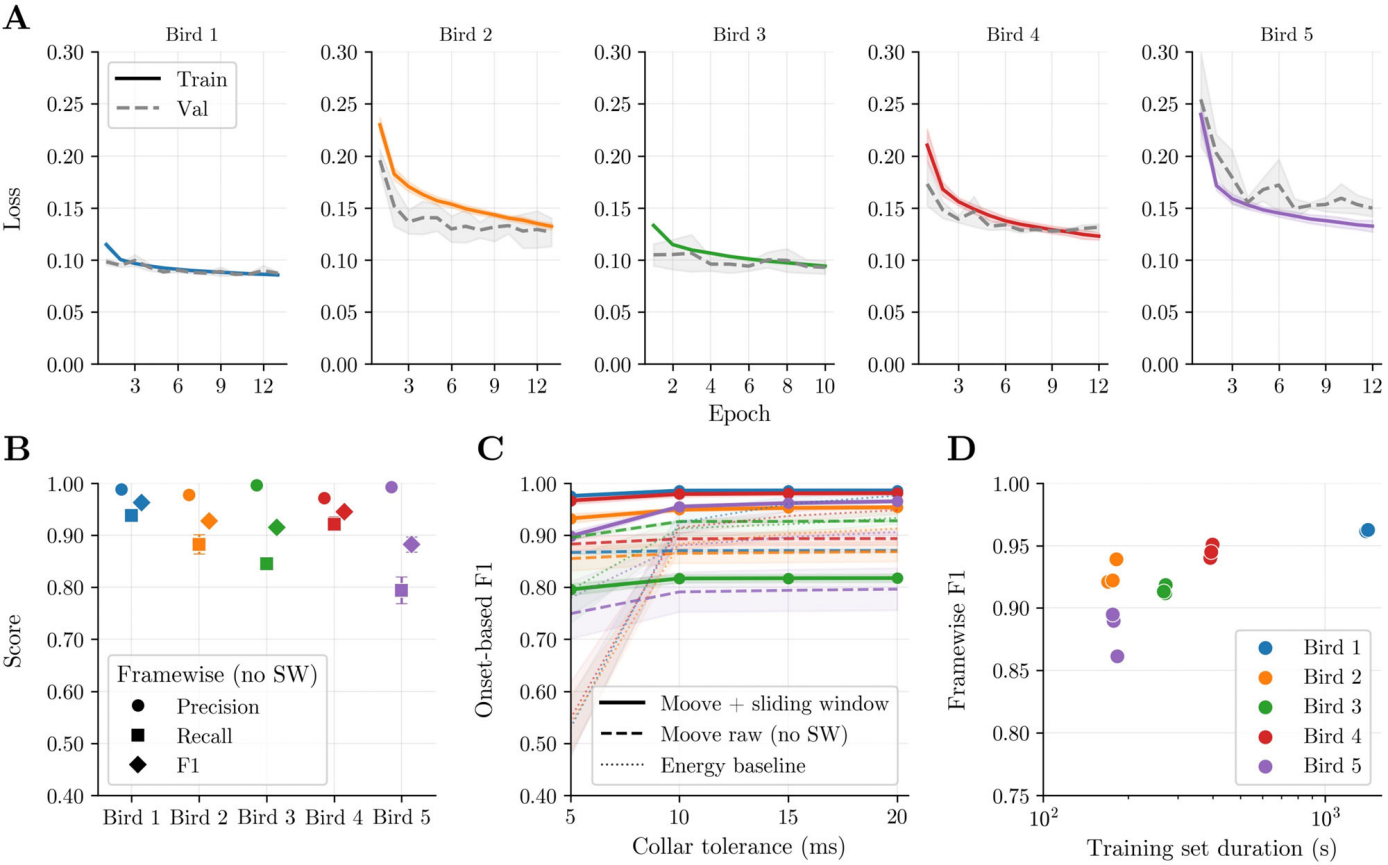
Segmentation network performance across five birds (3 training replicates each). ***A***, Training (colored, solid) and validation (gray, dashed) loss curves for each bird, showing smooth convergence. Shaded regions indicate ±1 SD across replicates. Loss curves are truncated to the shortest replicate per bird so that mean ± SD are computed over the same number of epochs. ***B***, Framewise precision, recall, and *F*1 score (raw network output after threshold, no sliding window postprocessing) for each bird. Error bars show ±1 SD across replicates. ***C***, Onset-based segment-level *F*1 score as a function of collar tolerance (5–20 ms). Solid lines show Moove with sliding window postprocessing applied; dashed lines show Moove raw network output without sliding window postprocessing; dotted lines show the energy-based baseline (evfuncs; Nicholson, 2021) with threshold optimized on the validation set. Shaded regions indicate ±1 SD across replicates. A predicted segment is counted as a true positive when its onset falls within ± collar millisecond of a ground-truth onset. ***D***, Training set duration (seconds, log scale) versus framewise *F*1 score (raw, no sliding window postprocessing) for individual replicates, showing the relationship between training data size and segmentation performance.

**Figure 5.**
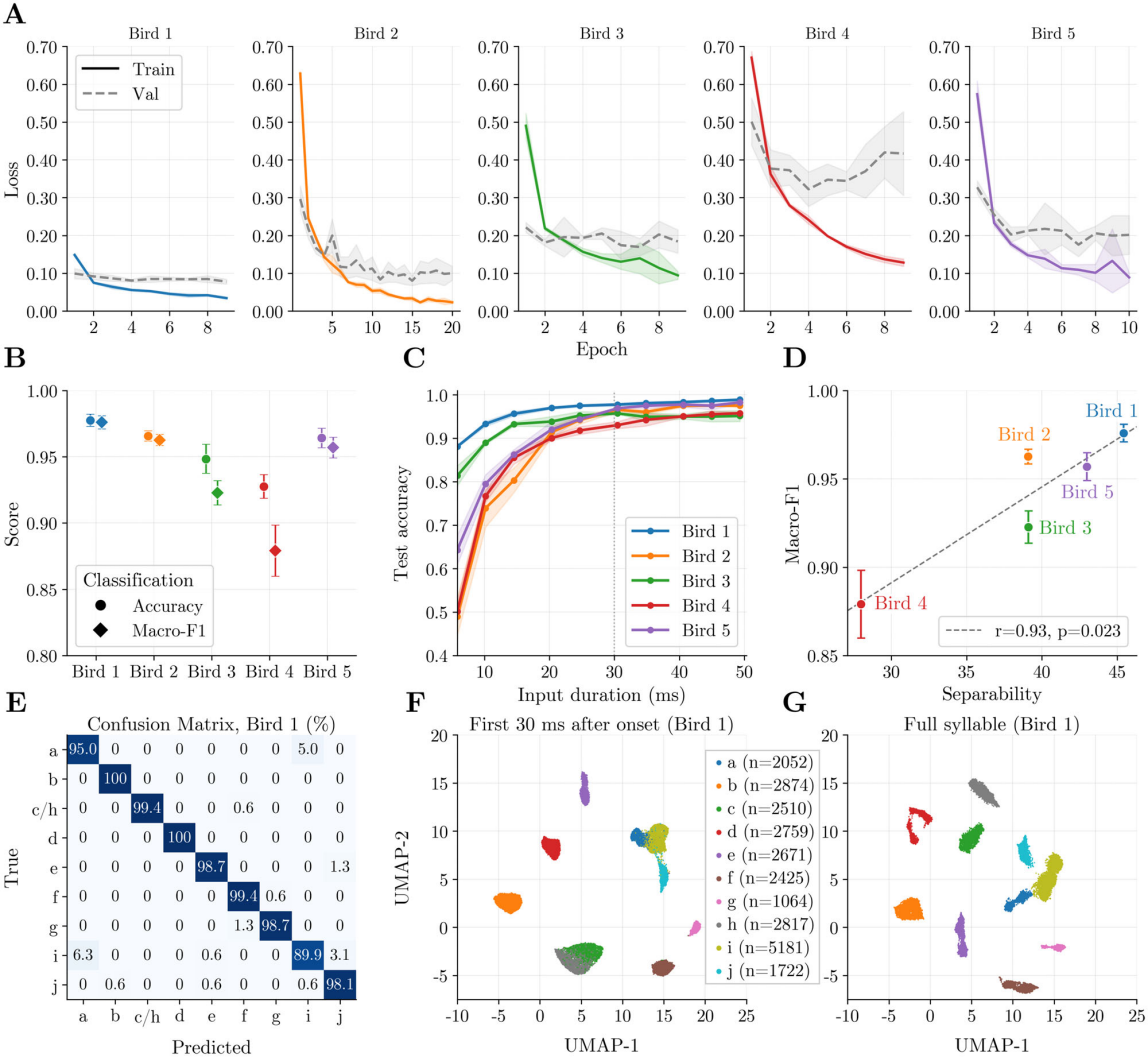
Classification network performance across five birds (3 training replicates each). Networks were trained on syllable spectrograms from the first 30 ms after onset. ***A***, Training (colored, solid) and validation (gray, dashed) loss curves for each bird, showing smooth convergence. Shaded regions indicate ±1 SD across replicates. ***B***, Classification accuracy and macroaveraged *F*1 score (dot plot with error bars) for each bird. Error bars show ±1 SD across replicates. ***C***, Effect of input duration on test accuracy for each bird for input windows from 5 to 50 ms after syllable onset. Shaded regions indicate ±1 SD across replicates. ***D***, Cluster separability (mean intercentroid distance in PCA space) versus macroaveraged *F*1 score across birds. The dashed line shows linear regression with Pearson’s *r* and *p* value. Error bars on macro-***F***1 indicate ±1 SD across replicates. ***E***, Confusion matrix for Bird 1 from the learning-experiment classifier (9 classes; syllables c and h merged as c/h). Numbers indicate row-normalized percentages. ***F***, UMAP projection of spectrograms using only the first 30 ms after syllable onset (Bird 1). Each point represents one syllable, colored by type. *N* indicates sample sizes for each syllable type. ***G***, UMAP projection of full-length spectrograms (normalized duration) for the same bird and same syllables as in ***F***.

Using networks trained on each bird's individual song repertoire, we characterized several aspects of Moove's performance. Across five birds, we evaluated segmentation and classification performance (Figs. 4, 5). To quantify the acoustic separability of each bird's syllable repertoire (Fig. 5*D*), we introduced a separability metric: syllable spectrograms were constructed using the same preprocessing pipeline as training (first 30 ms after onset, *z*-normalized per sample), projected onto 50 principal components (PCA), and the mean pairwise Euclidean distance between all per-class centroids in this reduced space was used as a separability measure.

**Figure 6.**
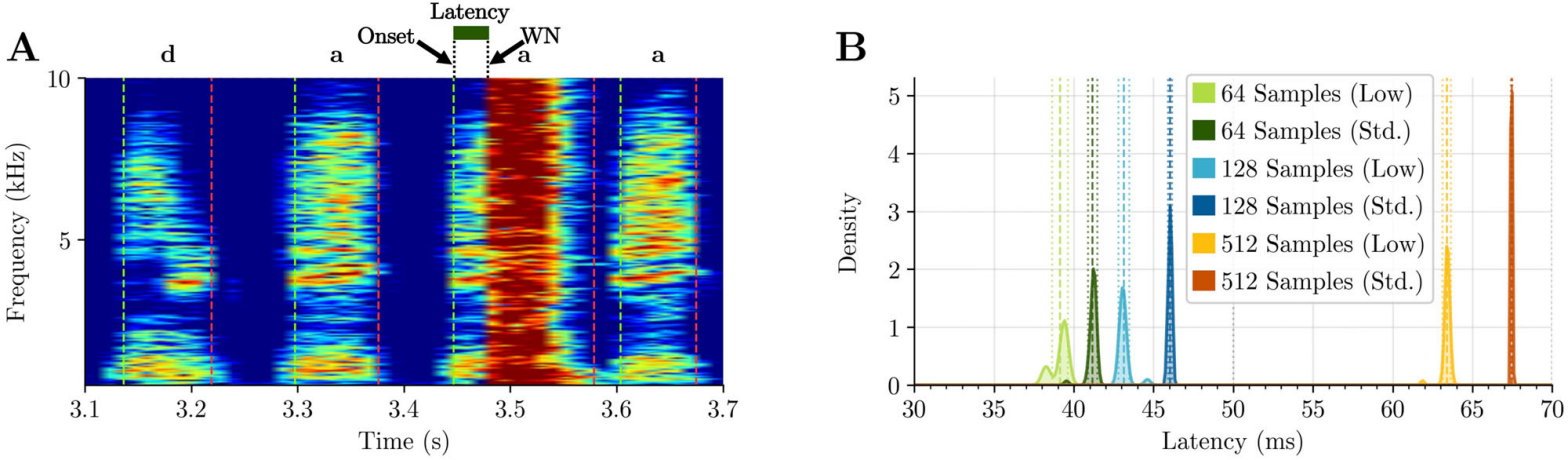
Latency characterization across audio buffer configurations. ***A***, Example spectrogram showing latency measurement in Bird 2. Green vertical dashed lines mark detected syllable onsets, red dashed lines mark detected offsets. In this example, the second occurrence of syllable “a” within a repeat phrase was targeted with WN playback. Latency (green bar) is measured from the onset of the targeted syllable to the onset of WN. ***B***, Distribution of measured latencies for six audio buffer configurations. Three buffer sizes (64, 128, 512 samples) tested with two driver modes (Low Latency or Standard) using the Yamaha Steinberg USB Driver on Windows. Each distribution represents ≥50 triggered events.

**Figure 7.**
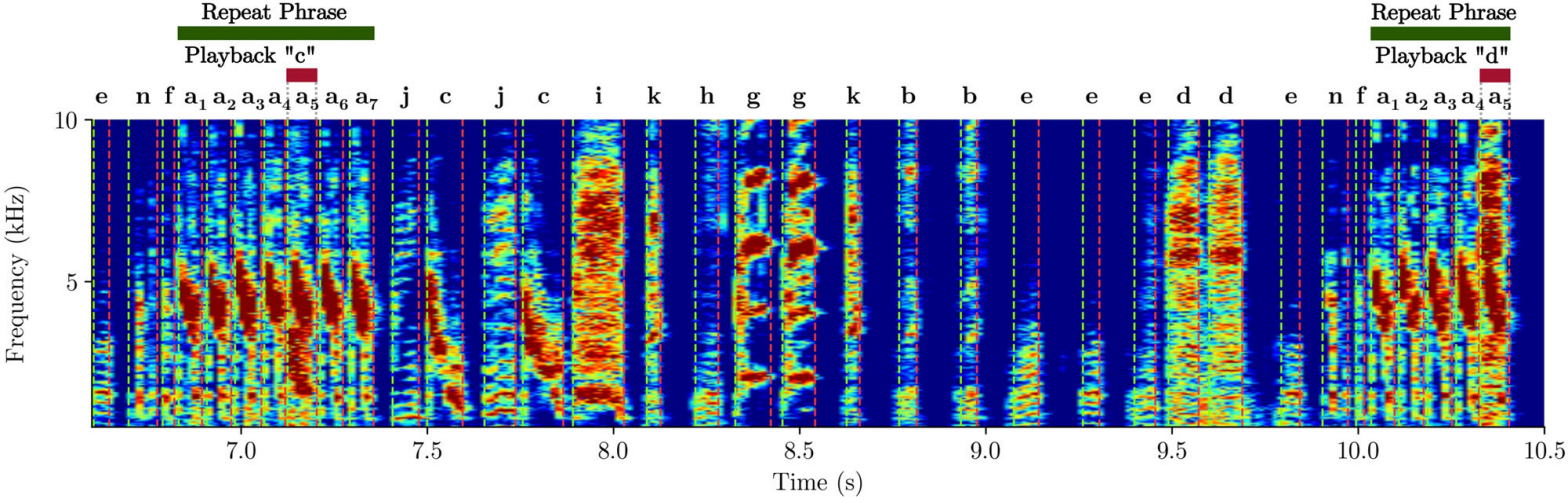
Repeat targeting with position-specific auditory feedback. Example bout demonstrating targeting of the fourth syllable in a repeat phrase of syllable “a” in Bird 3. Green bars indicate repeat phrase “a.” Red bars indicate auditory playback of different auditory stimuli (syllables, “c” and “d”). Vertical dashed lines mark detected syllable onsets. Latency was set so that playback overlaps syllable a_5_.

In two birds, we further characterized system latency and tested position-specific repeat–targeting capabilities. To characterize latency, we targeted a fixed syllable sequence and measured the time interval between syllable onset detection and WN playback (Fig. 6). We tested different audio buffer sizes (64, 128, and 512 samples) and mode settings using the Yamaha Steinberg USB Driver on Windows. For each configuration, we collected at least 50 triggered instances to obtain reliable latency distributions. To demonstrate the ability to target specific repetitions within repeat phrases, we targeted the fifth occurrence of syllable “a” and delivered position-specific auditory feedback by playing back different auditory stimuli (syllables “c” or “d”) for different instances (Fig. 7).

### Experiment 2: sequence modification learning experiment

A Bengalese finch with a clear branch point in its song was used for the learning experiment. Five days of baseline song were recorded with real-time processing disabled. These data were used to train the segmentation and classification networks. The trained models were then used for 4 consecutive days of WN training, targeting a specific syllable sequence to reduce its occurrence after the branch point. An 80 ms WN stimulus was played to overlay the targeted syllable.

Catch trials (10%) were included where no feedback was given. In this earlier version of Moove, catch trials were mistakenly only assigned to bouts that contained the target sequence. Therefore, transition probabilities computed from catch-trial bouts alone may overestimate the occurrence of the target transition. To correct for this sampling bias, we performed a resampling procedure: we randomly sampled 10% of all bouts and replaced any noncatch-trial bouts containing the target sequence with randomly drawn catch-trial bouts (with replacement); any noncatch-trial bouts that did not contain the target sequence also did not contain WN and could therefore be treated like catch trials. We repeated this resampling for 1,000 iterations and from the resulting distribution, we extracted minimum, median, and maximum transition probabilities. This simulates the effect that the proper assignment of catch trials to all bouts would have had on the analysis.

Transition probabilities were calculated as the proportion of transitions from “h–c” to each following syllable across all bouts during baseline and postscreening periods, and in resampled catch-trial bouts during the training period.

Two postscreening sessions were conducted 16 and 37 d after the last training day, respectively, to assess the persistence of learning. The bird was housed in a group aviary between baseline, training, and postscreening sessions.

## Results

We developed Moove, a neural network-based system for real-time segmentation and classification of Bengalese finch syllables. Moove uses a dedicated network for syllable segment detection and a second network for classification of syllable types using only the first 30 ms of acoustic information after syllable onset, thereby achieving syllable identification and targeting during ongoing syllables.

### Segmentation performance

We tested segmentation performance on five male Bengalese finches with different songs. Segmentation networks were trained separately for each bird to distinguish syllable segments from gaps in the audio stream. All network hyperparameters corresponded to the Moove default configuration. To assess robustness, we trained three replicates per bird with different random seeds for data splitting; all metrics are reported as mean ± standard deviation across replicates. Training data ranged from 113 to 573 manually corrected bouts across birds (mean, 236 ± 172 bouts), corresponding to 176–1,407 s of audio, which were randomly split into training (70%), validation (15%), and test (15%) subsets (see Table 1 for individual bird data). Across all birds, training and validation loss curves converged smoothly (Fig. 4*A*), with early stopping terminating training after 10–24 epochs (mean, 15 ± 4 epochs). Framewise precision, recall, and *F*1 scores were computed on each bird's test set from the raw network output after thresholding, without sliding window postprocessing (Fig. 4*B*), yielding mean *F*1 scores ranging from 0.882 to 0.962 across birds (Table 1). The sliding window postprocessing algorithm improved frame-level *F*1 only modestly, whereas its main benefit was observed for onset-based segment detection, the operationally relevant measure for real-time targeting (Fig. 4*C*; Table 1). These results indicate that the network makes accurate predictions when distinguishing syllable segments from gaps. Training set duration was positively associated with framewise *F*1 (Fig. 4*D*): the bird with the most training data (1,407 s) achieved the highest F1 (0.962), while birds trained on only 176–394 s of audio ranged from 0.882 to 0.945.

**Table 1.** Segmentation network performance across five birds

Bird	#Syl	Dur (s)	Ep	FW-P	FW-R	FW-F1	Col@10 ms	Col@20 ms
Bird 1	18,321	1,407.0	16	0.988 ± 0.001	0.938 ± 0.002	0.962 ± 0.001	0.986 ± 0.000	0.986 ± 0.000
Bird 2	3,772	175.7	18	0.978 ± 0.006	0.882 ± 0.019	0.928 ± 0.008	0.949 ± 0.003	0.954 ± 0.005
Bird 3	7,271	269.6	14	0.996 ± 0.001	0.845 ± 0.005	0.914 ± 0.003	0.817 ± 0.010	0.818 ± 0.009
Bird 4	7,359	393.7	14	0.971 ± 0.008	0.921 ± 0.014	0.945 ± 0.004	0.979 ± 0.006	0.981 ± 0.005
Bird 5	3,138	178.9	12	0.993 ± 0.003	0.794 ± 0.025	0.882 ± 0.015	0.955 ± 0.003	0.965 ± 0.003

Dur, training set duration in seconds; Syl, number of syllables in training set; Ep, training epochs; FW-P/R/F1, framewise precision, recall, and *F*1 score; Col@10/20, onset-based segment-level *F*1 with 10 and 20 ms collar tolerance (with sliding window postprocessing). All metrics reported as mean ± SD across three replicates.

To evaluate segment-level detection performance as deployed in real-time operation, we applied the sliding window postprocessing algorithm (see Materials and Methods) to the raw network predictions and computed onset-based segment-level precision, recall, and *F*1 with collar tolerances of 5, 10, 15, and 20 ms (Fig. 4*C*, solid lines; Table 1). This means that a predicted segment was counted as a true positive when its onset fell within ± collar millisecond of a ground-truth syllable onset, regardless of offset timing. This onset-only criterion reflects the primary requirement for closed-loop targeting, where reliable onset detection determines accurate classification and feedback timing. By default, Moove processes audio in 64-sample frames at 44.1 kHz, yielding a frame-level temporal resolution of 1.45 ms. Onset decisions are then refined by the sliding window postprocessing step (see Materials and Methods). With a 10 ms collar, onset-based F1 scores with sliding window postprocessing ranged from 0.949 to 0.986 for four of the five birds, with one clear outlier, Bird 3 (Table 1). Bird 3 had only 0.817 F1 due to a mismatch between the bird-specific classification threshold and the standard sliding window parameters, which caused many true onsets to be discarded. We retrained the segmentation network for Bird 3 with the “Overlap chunks” option enabled during training dataset creation, which increases training sample density by advancing the input window one audio chunk at a time instead of by the full input-window length. This improved onset-based segmentation F1 to 0.917.

To benchmark segmentation performance, we compared Moove against an energy-based segmentation baseline (evfuncs; Nicholson, 2021; Fig. 4*C*, dotted lines; Table 1). For each bird, both the baseline amplitude threshold and Moove's sliding window parameters were optimized by grid search on the validation set. The energy-based baseline operates on a smoothed amplitude envelope (smoothing window, 2 ms) and detects onsets as threshold crossings of this envelope. Moove outperformed the energy baseline in onset collar F1 at 10 ms for four of five birds (Bird 1, +0.06; Bird 2, +0.07; Bird 4, +0.07; Bird 5, +0.08). For Bird 3, the standard Moove result fell below the energy-based baseline (0.913), but after retraining with improved parameters (see above), performance was slightly above baseline (+0.004).

To further quantify segmentation accuracy, we computed the signed onset time difference (predicted minus annotated) for all matched segments (true positives at 10 ms collar), using the held-out test set of each replicate. Across all birds and replicates (*n* = 24,277 matched onsets), mean onset error was −0.5 ± 1.9 ms (median, −1.5 ms), confirming that detected onsets closely match the annotated boundaries. The median of −1.5 ms corresponds to approximately one audio chunk, consistent with the frame-level resolution of the network. The network's inference speed was ∼0.73 ± 0.1 ms per audio chunk when run with an inference batch size of 1 on a standard CPU, enabling real-time processing at the 44.1 kHz sampling rate.

### Classification performance

Classification networks were trained for each of the five male Bengalese finches to classify syllable types based on spectrograms computed from the first 30 ms after syllable onset (Fig. 5). All network hyperparameters corresponded to the Moove default configuration. As for segmentation, three replicates per bird were trained with different random seeds for data splitting; all metrics are reported as mean ± standard deviation across replicates. Training data ranged from 3,146 to 18,335 syllables (96–559 s) across birds, which were randomly split into training (70%), validation (15%), and test (15%) subsets (see Table 2 for individual bird data). Training and validation loss converged smoothly for all birds (Fig. 5*A*). Across birds, test accuracies ranged from 0.927 to 0.977, with macroaveraged F1 scores ranging from 0.879 to 0.976 (Table 2; Fig. 5*B*). Early stopping with a patience of 5 epochs prevented overfitting, with training terminating after 9–33 epochs (mean, 15 ± 6 epochs). The network's inference speed was ∼0.94 ± 0.25 ms per spectrogram when run with an inference batch size of 1, enabling real-time classification in a separate processing thread.

**Table 2.** Classification network performance across five birds

Bird	#Cls	#Syl	Dur (s)	Ep	Accuracy	Mac-P	Mac-R	Mac-F1
Bird 1	9	18,335	558.7	11	0.977 ± 0.005	0.972 ± 0.006	0.981 ± 0.004	0.976 ± 0.005
Bird 2	10	3,803	115.9	24	0.966 ± 0.004	0.956 ± 0.010	0.972 ± 0.007	0.963 ± 0.004
Bird 3	11	7,115	216.8	12	0.948 ± 0.011	0.913 ± 0.011	0.945 ± 0.010	0.923 ± 0.009
Bird 4	8	7,360	224.3	10	0.927 ± 0.009	0.863 ± 0.023	0.907 ± 0.017	0.879 ± 0.019
Bird 5	8	3,146	95.8	13	0.964 ± 0.007	0.954 ± 0.009	0.963 ± 0.004	0.957 ± 0.008

Cls, number of syllable classes; Syl, number of syllables in training set; Dur, training set duration in seconds (based on 30.47 ms input window per syllable); Ep, training epochs; Acc, test accuracy; Mac-P/R/F1, macroaveraged precision, recall, and *F*1 score. All metrics reported as mean ± SD across three replicates.

To assess the trade-off between classification accuracy and input duration, we tested classification performance across input durations from ∼5–50 ms for each bird (Fig. 5*C*). Accuracy increased steeply up to 30 ms across all birds and showed only modest improvements thereafter. Even at very short input durations (5.8 ms; corresponding to four input chunks), networks achieved 48.9–88.1% accuracy across birds, demonstrating substantial discriminability from syllable onsets alone. At 30 ms input duration, 96% of the total accuracy gain observed (relative to the tested 5–50 ms range) was already captured, with only 1.1 percentage points of additional accuracy remaining at longer durations (Table 3). Based on these results, we selected 30 ms as the default input duration for Moove, balancing high accuracy with low latency for real-time experiments. This parameter can be adjusted depending on the specific experimental requirements and song structure.

**Table 3.** Mean test accuracy across five birds as a function of input duration

Input duration (ms)	Mean accuracy	Δ acc/ms	% of total gain
5.8	0.666 ± 0.162	—	0%
10.2	0.825 ± 0.078	+0.036/ms	52%
14.5	0.882 ± 0.058	+0.013/ms	71%
20.3	0.928 ± 0.027	+0.008/ms	86%
24.7	0.946 ± 0.020	+0.004/ms	92%
30.5	0.960 ± 0.017	+0.002/ms	96%
34.8	0.962 ± 0.017	+0.000/ms	97%
40.6	0.967 ± 0.015	+0.001/ms	99%
45.0	0.968 ± 0.016	+0.000/ms	99%
49.3	0.971 ± 0.016	+0.001/ms	100%

Δ acc/ms, marginal accuracy gain per millisecond of additional input. % of total gain, cumulative gain relative to the total achievable gain from 5.8 to 49.3 ms. Mean ± SD across 5 birds × 3 replicates.

We computed a cluster separability score for each bird (see Materials and Methods) to confirm that the acoustic structure of the syllable repertoire explains variation in classification accuracy (Fig. 5*D*). For Bird 1, which was used in the subsequent learning experiment, we merged syllables “c” and “h” because they were spectrally similar within the first 30 ms and occurred in a fixed sequence order, so distinguishing them was not required for online sequence targeting. This improved syllable classification accuracy on the remaining syllables without affecting the outcome of the learning experiment (Fig. 5*E*). The confusion matrix for this bird shows that most syllable types were classified with >98% accuracy. Lower accuracies occurred for syllables with similar spectral structure in the first 30 ms: syllable “i” tended to be confused with “a” (6.3%) and “j” (3.1%), while “a” was misclassified as “i” in 5% of the cases. These confusions reflect genuine spectral similarity in the early portions of these syllables, as visualized in the UMAP projection based on the first 30 ms (Fig. 5*F*; clusters for “i,” “j,” and “a,” as well as “c”/”h,” show substantial overlap). In contrast, when using full-length syllables, all syllable types form well-separated clusters (Fig. 5*G*), confirming that the classification errors stem from limited temporal information rather than inherent ambiguity.

### Latency characterization

A critical requirement for effective operant conditioning is low-latency feedback delivery (Tumer and Brainard, 2007; Charlesworth et al., 2011)*.* To characterize Moove's latency, we measured the time interval between the detected syllable onset and the onset of WN playback (Fig. 6*A*). We targeted a fixed syllable sequence in Bird 2 and measured latency for each triggered event with different audio buffer configurations.

For the audio interface in our setup, we used the Yamaha Steinberg USB Driver on Windows and tested the “Low Latency” and “Standard” driver modes with adjustable buffer sizes. Larger buffer sizes provide more stable audio playback by reducing the risk of buffer underruns (audio dropouts) but increase latency. We systematically tested playback latency with three buffer sizes (64, 128, and 512 samples) with the two driver modes (Fig. 6*B*). Buffer size had a substantial effect on latency: 64-sample buffers produced the shortest latencies (mean, 39.14 ± 0.49 ms in Low Latency mode, 41.18 ± 0.30 ms in Standard mode), while 512-sample buffers resulted in longer latencies (mean, 63.39 ± 0.26 ms in Low Latency mode, 67.45 ± 0.05 ms in Standard mode). Driver mode also affected latency, with Low Latency mode reducing mean latency by ∼6.01 ± 11.64 ms compared with Standard mode across all buffer sizes. For each configuration, we collected at least 50 triggered instances to obtain reliable latency distributions (*n* = 60–231 per condition).

### Low-latency feedback performance of Moove

With optimized settings (64-sample buffer, Low Latency mode), mean feedback latencies were 39.14 ± 0.49 ms. Given that Moove waits 30 ms after syllable onset to gather acoustic information for classification, the remaining system overhead (hardware, audio processing, playback) accounts for only ∼10 ms of the latency. This low-latency performance is suitable for operant conditioning experiments where temporal contingency between behavior and feedback is critical.

### Repeat targeting with position-specific feedback

To study behavioral influences on syllable repetition, we were particularly interested in targeting individual instances of the same syllable within repeat phrases (Binwal and Veit, 2026). For example, we might want to target only the fifth occurrence of syllable “a” in a phrase consisting of repetitions of syllable “a” (Fig. 7). Repeat phrases can be particularly challenging for template-based approaches, which match templates on a continuous input stream without segmentation. They require setting parameters for repetitions of template matches, with refractory periods after matches ensuring that there can be no other matches within the same syllable. Due to subtle changes in the timing of syllables and gaps throughout the repeat phrase, it can be challenging or impossible to define templates and refractory periods in a way that they do not become misaligned over the duration of the phrase, eventually leading to counting errors. Moove's syllable-based approach eliminates this source of counting errors and ensures that latency remains stable with respect to syllable onsets throughout the phrase. As in other sequences, the target can be chosen through pattern matching of regular expressions, allowing the user to specify which position(s) to target within the repeat phrase. As Moove explicitly detects syllable onsets and saves the history of previously detected syllables, it eliminates the need to discriminate spectrally similar syllables based on timing alone. As an illustration of this use case, we show an example song bout in which we targeted the fifth repetition of syllable “a” within a repeat phrase for auditory feedback manipulations (Fig. 7). Moove was set to trigger playback by detecting the onset of the fourth “a” (a4), and playback latency was chosen so that the playback stimulus overlaps with the fifth “a” (a5). The example bout shows two phrases in which Moove detected a4 and triggered playback of different auditory stimuli (“c” and “d”; red bars). Within this example experiment, Moove correctly triggered on 34 of 36 target phrases, with one false positive (precision, 97.1%; recall, 94.4%; *F*1 = 0.96), demonstrating its usefulness for targeting specific positions within repeat phrases.

### Sequence modification learning experiment

**Figure 8.**
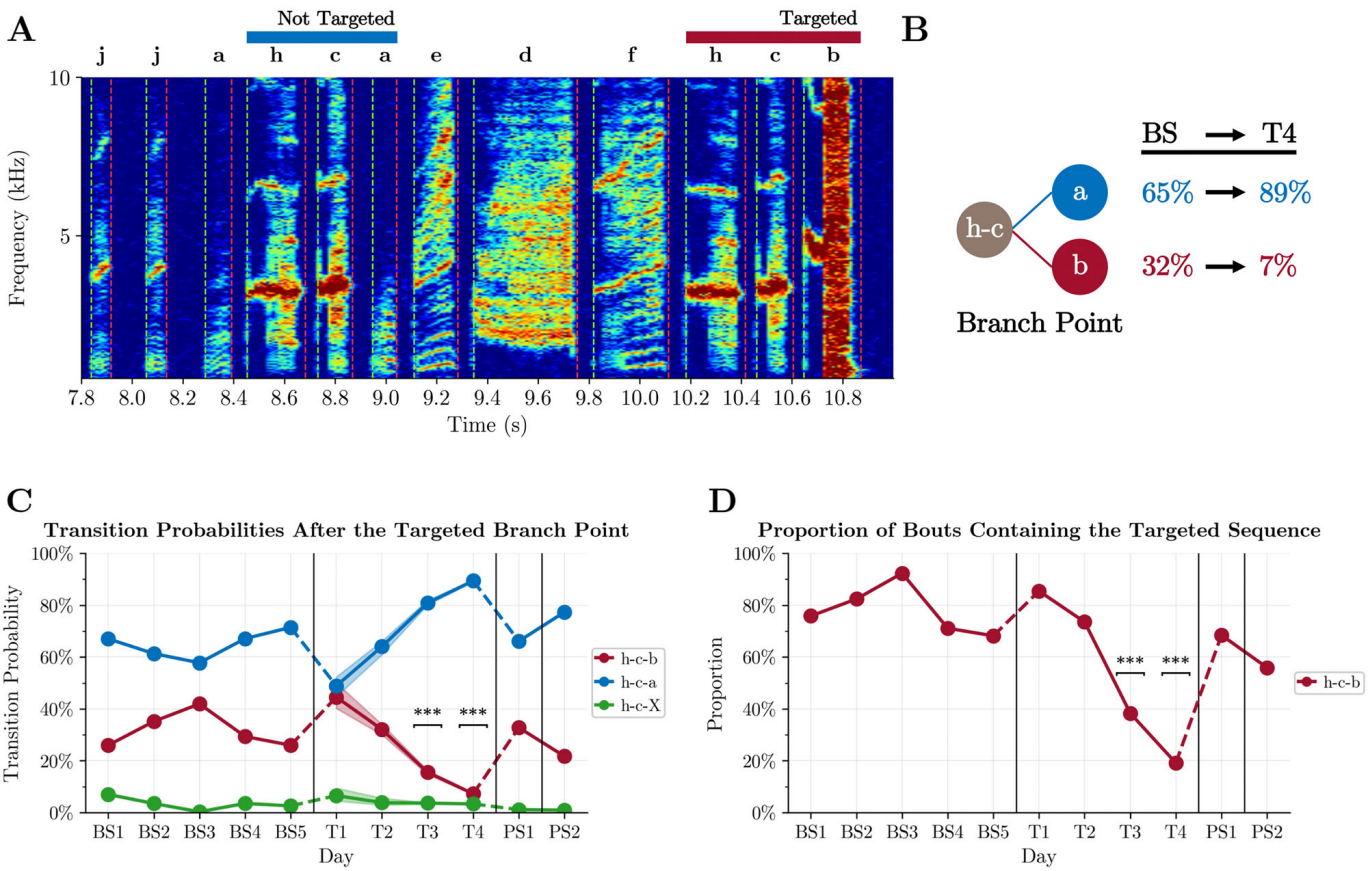
Sequence modification learning experiment using WN feedback. ***A***, Example spectrogram from a training day in Bird 1 showing selective targeting. Syllable labels indicate detected types online. Left, Sequence “h–c–a” (blue bar), not targeted. Right, Sequence “h–c–b” (red bar) followed by WN playback over syllable “b.” Vertical dashed lines mark detected syllable onsets and offsets. ***B***, Summary diagram of learning outcome. Left, Mean baseline (BS1–BS5) transition probabilities from “h–c” to “a” (blue, 65%) and “b” (red, 32%). Right, After training (T4), probabilities shifted to 89% for “a” and 7% for “b”. ***C***, Transition probabilities across experimental days. Red, “h–c–b” (targeted, median values); blue, “h–c–a” (main alternative branch); green, other rare transitions. Shaded regions show min–max range from catch-trial adjusted resampling (1,000 iterations). Vertical lines indicate noncontinuous days between baseline (BS1–BS5), training (T1–T4), and postscreening (PS1–PS2) periods. Statistical significance: *** *p* < 10^−12^ (two-proportion *z*-test). ***D***, Proportion of bouts containing the target sequence “h–c–b” across days. Statistical significance as in ***C***.

To test whether Moove allows effective operant conditioning, we conducted a sequence modification learning experiment in Bird 1. This bird exhibited a branch point following the syllable sequence “h–c” during baseline recordings (Fig. 8*A*), singing either “h–c–a” in 65% or “h–c–b” in 32% of the instances, averaged across the baseline days (Fig. 8*B*). We chose to target syllable sequence “h–c–b” with WN feedback, which typically leads to a reduction of the target sequence over multiple training days (Warren et al., 2012; Veit et al., 2021; Fortkord and Veit, 2025). The example spectrogram (Fig. 8*A*) illustrates the selective targeting of one branch: the sequence “h–c–a” (blue bar) was correctly not targeted, while “h–c–b” (red bar) was accurately detected and the rest of syllable “b” covered by a WN playback stimulus. To disentangle learning-related changes from acute effects of WN on subsequent song, 10% of detected sequences were designated as catch trials where no WN was played despite detection of the target sequence. In this experiment using an earlier version of Moove, catch trials were therefore only assigned to bouts that contained the target sequence. This differs from the standard definition (Tumer and Brainard, 2007; Warren et al., 2012), where catch trials are randomly sampled from all bouts regardless of content. The implementation has subsequently been updated in Moove to use the standard definition. To correct for this sampling bias, we performed a resampling procedure for analyses (see Materials and Methods).

Training with WN feedback resulted in clear behavioral changes. By the final training day (T4), transition probabilities at the target branch point had shifted from the baseline average: transitions to “a” increased to 89% while transitions to “b” decreased to 7% (Fig. 8*B*), demonstrating successful sequence modification which reduced the number of WN feedbacks. Figure 8*C* shows the full experimental timeline from baseline across all training days and postscreening days. In line with the steady reduction in the target branch over the training days (T1–T4), the bird increased the alternative branch “a” after “h–c” (blue line). Shaded regions show the range between minimum and maximum values from the resampling. Statistical analysis using two-proportion *z*-tests on both median and maximum values (representing the most conservative estimate) confirmed that from training Day T3 onward, the reduction in “h–c–b” transitions was highly significant compared with baseline (median, T3 *p* = 3.24 × 10^−20^; T4 *p* = 1.73 × 10^−12^; maximum, T3 *p* = 3.17 × 10^−18^; T4 *p* = 1.73 × 10^−12^). The proportion of bouts containing the targeted sequence “h–c–b” also decreased dramatically, from 70 to 85% during baseline to 19% on Day T4 (Fig. 8*D*), with highly significant reductions from Day T3 onward (T3, *p* = 1.87 × 10^−14^; T4, *p* = 1.78 × 10^−24^). In postscreening sessions conducted after training, transition probabilities and target sequence occurrence had partially recovered toward baseline (PS1 vs baseline, *p* = 1.08 × 10^−5^).

Throughout the experiment, Moove performed reliably: zero false-positive WN triggers occurred across all four training days. False negatives (missed triggers) were <1%. In this experiment (performed before the optimization of audio driver parameters shown in Fig. 6), mean WN onset latency measured from the onset of syllable “b” was 90.87 ± 11.04 ms (*n* = 315 triggers). These results demonstrate that Moove enables accurate syllable targeting suitable for reinforcement learning experiments, with behavioral outcomes comparable to those achieved using template-based real-time systems.

## Discussion

We developed Moove, a two-stage neural network system that enables real-time syllable targeting during ongoing birdsong. The name is derived from “Japanische Mövchen,” the German name for Bengalese finches. Moove achieved high segmentation and classification accuracy with low feedback latencies. A sequence modification experiment validated its effectiveness for closed-loop operant conditioning. Moove achieves feedback delivery before syllable offset, a capability previously limited to template-based systems, while offering advantages in syllable coverage, classification accuracy, and experimental flexibility.

### Moove can target ongoing syllables

The central innovation is classifying and targeting syllables using neural networks before their acoustic offset, addressing a critical limitation in current closed-loop tools. Existing real-time neural network systems for birdsong classification (Steinfath et al., 2021; Schulthess et al., 2023; Kawaji et al., 2024) determine syllable identity only after detecting syllable offset. Feedback delivery during ongoing vocalizations is critical for many operant conditioning experiments, where temporal contingency between behavior and outcome affects learning efficacy (Tumer and Brainard, 2007; Charlesworth et al., 2011). Other protocols requiring feedback during the target syllable include auditory feedback manipulation or song-triggered optogenetic stimulation.

Moove's approach can be understood within the broader frameworks of voice activity detection (Hughes and Mierle, 2013) and sound event detection (Stowell, 2022; Kershenbaum et al., 2025), applied to predicting sequences of animal sounds (Kershenbaum et al., 2016). Frame classification models like TweetyNet (Cohen et al., 2022) and DAS (Steinfath et al., 2021) classify each acoustic frame and achieve high offline accuracy through extensive temporal context. TweetyNet uses recurrent layers, which introduce computational overhead and make it primarily designed for offline annotation. With smaller audio chunks, DAS has been used for closed-loop classification of Drosophila song types, but we are not aware of studies using DAS for online birdsong annotation so far. SAIBS (Kawaji et al., 2024) uses threshold-based spectral change detection followed by classification on long temporal windows, which likely include preceding syllables. In the experiment by Kawaji et al., detected syllables were accumulated into a string on which target patterns were matched after bout end, so that feedback is delivered with substantial latency at the bout level rather than during individual syllables. More recently, transformer-based approaches such as WhisperSeg (Gu et al., 2024) have been applied to animal sound segmentation but are typically orders of magnitude larger, making real-time deployment challenging. Our approach relies on the effectiveness of lightweight binary classifiers for sound segmentation in bioacoustics (Baggi et al., 2023). TinyBird-ML (Schulthess et al., 2023) similarly uses separate networks for real-time detection and classification but is designed for deployment on wearable sensors with constraints on power and temporal resolution.

To fill the gap between these approaches and template-based real-time systems, Moove uses a forward-only architecture with a small temporal window after onset detection. It prioritizes temporal precision for real-time targeting while maintaining sufficient accuracy. Moove can therefore classify ongoing syllables while they are still being sung, enabling feedback delivery before syllable offset.

### Moove classifies syllables using only local acoustic information

Syllables in Moove are classified based solely on their own acoustic properties, without incorporating information about preceding syllables. While incorporating sequential context could dramatically improve accuracy by exploiting probabilistic sequence structure, this would introduce systematic bias against detecting rare or novel syllable transitions. For experiments investigating vocal sequencing or designed to modify sequences through operant conditioning, context-independent classification is essential. A context-dependent classifier would become progressively less accurate as birds learn to avoid targeted sequences, requiring retraining during experiments or obscuring sequence modifications through mislabels based on sequential expectations of the classifier.

### Moove performs syllable-based classification and can target syllables based on sequence context

Template-based systems like EvTAF (Tumer and Brainard, 2007; Roberts et al., 2012; Ali et al., 2013; Xiao et al., 2018; Riedner and Adam, 2020; Moll et al., 2023; Trusel et al., 2025) recognize syllables by matching a short audio segment to a spectral template, achieving very low latencies when combined with specialized hardware. However, template creation is labor-intensive, requiring manual design and iterative optimization for each target syllable. Even with optimization algorithms, only 18–43% of syllables in Bengalese finch repertoires can be detected reliably using manually created templates (Kulkarni and Troyer, 2020), which fundamentally limits experimental accessibility. When birds modify their syllables during learning experiments, fixed templates become less effective and require manual updating.

Defining context-dependent targets compounds these challenges, requiring complex combinations of templates and timing constraints, with logic operators that can become unwieldy as sequence complexity increases. Because Moove explicitly identifies each syllable as it occurs, experimenters can easily target syllables based on their identity and the identity of preceding syllables (“sequence context”). This syllable-based approach proves particularly valuable for Bengalese finch song, where acoustically identical syllables can appear in multiple sequence contexts (such as “d-e_1_-f” or “x-e_2_-y”; Jin and Kozhevnikov, 2011; Katahira et al., 2011; Veit et al., 2021; Koparkar et al., 2024; Lu et al., 2025). Experimenters can specify even complex target sequences by listing the syllable labels with regular expression-based pattern matching. A template-based approach is particularly difficult for repeat phrases, where subtle changes in syllable and gap timing throughout the phrase cause template-timing combinations to become misaligned, eventually leading to counting errors. Moove's syllable-based approach is intuitive to use and accommodates natural variability in song structure and timing. It eliminates timing-based counting errors in repeat phrases and ensures stable feedback latencies relative to syllable onsets (Fig. 7). Syllable-based detection also reduces false-positive triggers from call vocalizations or cage noise, which can spuriously match acoustic templates.

### Moove does not require specialized hardware or skills

Moove's Python-based design facilitates modification and extension, enabling researchers to adapt the system to specific experimental needs. Moove's source code is released under the MIT License. The system operates on standard desktop recording hardware with commodity audio interfaces. File formats and annotation structures maintain compatibility with existing workflows using EvTAF, facilitating adoption by research groups transitioning from these systems. Together, MooveTAF and MooveGUI provide a complete pipeline from data collection through network training, where researchers can train bird-specific models without deep machine learning expertise. The semiautomated approach integrates energy-based segmentation and unsupervised clustering with manual tools for correction and refining cluster memberships. The modular architecture permits researchers to modify segmentation and classification components separately for specific experimental needs.

### Moove is suitable for reinforcement learning experiments

The sequence modification learning experiment validated Moove's effectiveness for closed-loop operant conditioning. After targeting one branch of a branch point with WN feedback, the bird exhibited a clear shift in transition probabilities from baseline. These learned changes are comparable to published sequence learning experiments (Warren et al., 2012; Veit et al., 2021; Fortkord and Veit, 2025). In this experiment, mean WN onset latency was ∼90 ms, as it was conducted before the audio driver optimizations that reduced latency to ∼40 ms in later testing. Despite this higher latency, the bird learned effectively, suggesting that this temporal contingency can be sufficient for operant conditioning in a sequence learning task. Our pilot results demonstrate that Moove enables accurate, reliable syllable targeting suitable for reinforcement learning experiments, with behavioral outcomes comparable to those achieved using template-based systems.

### Limitations and future perspectives

Several aspects of Moove's current implementation present opportunities for extension. The feedback latency of ∼40 ms proved sufficient for sequence learning but may require reduction for pitch learning or other experiments (Tumer and Brainard, 2007). As the latency is composed of the 30 ms default classification window and 10 ms system overhead, this could be achieved through shorter classification windows, at the cost of accuracy. This trade-off is user-adjustable without code modification. Hardware optimizations or more complex network architectures could potentially improve discrimination without extending the temporal window.

The segmentation network occasionally misidentifies cage noise as syllable segments, though this did not affect targeting in our experiment. Future implementations could include a dedicated noise class during training, as in other systems (Steinfath et al., 2021; Cohen et al., 2022).

Applicability of Moove to vocal signals in other species will depend on their vocal structure (Sainburg et al., 2020). Songbird species with discrete syllable repertoires (e.g., zebra finches) should be well captured by Moove's approach. Bird species with highly variable or continuous vocalizations (Markowitz et al., 2013; Cohen et al., 2020; Costalunga et al., 2023; Zhao et al., 2023) present greater challenges due to less discrete syllable boundaries and may require substantial modifications to segmentation and classification approaches. Extension to closed-loop experiments requiring the detection of vocalizations in other species (Hage et al., 2013; Pomberger et al., 2018; Liao et al., 2024) may be achievable with suitable adaptation.

Moove's real-time targeting capability supports diverse experimental applications beyond the operant conditioning protocol presented here. The system could be used for song-triggered manipulations of auditory feedback (Tumer and Brainard, 2007), visual feedback (Zai et al., 2020), somatosensory feedback (McGregor et al., 2022), neural microstimulation (Moll et al., 2023), optogenetic perturbations (Hisey et al., 2018), and access to conspecifics (Ben-Tov et al., 2023). Adapting Moove for these modalities would primarily require modifying the feedback output module, as the segmentation and classification pipeline is modality-independent. The open-source release will enable community-driven extensions and validation across diverse experimental contexts and species. For experimental protocols involving sequence targeting, Moove's combination of broad syllable coverage, high accuracy, flexible sequence specification, and moderate latency presents a practical solution for previously inaccessible experimental approaches.

## Synthesis

Reviewing Editor: David Franklin, Technische Universitat Munchen Decisions are customarily a result of the Reviewing Editor and the peer reviewers coming together and discussing their recommendations until a consensus is reached. When revisions are invited, a fact-based synthesis statement explaining their decision and outlining what is needed to prepare a revision will be listed below. The following reviewer(s) agreed to reveal their identity: David Nicholson. Note: If this manuscript was transferred from JNeurosci and a decision was made to accept the manuscript without peer review, a synthesis may not be available.

## Author Response

Reviewer comments:

Overall, I think this manuscript and the accompanying software fill a longstanding gap. Therefore I am in favor of publishing as quickly as possible instead of requiring any more experiments -- the entire community will benefit from the manuscript and software being made widely accessible as soon as possible. The reported results are more than sufficient for proof-of-concept, demonstrating the method performs as advertised, and additionally replicating a previous experiment using the new software.

All that said, I would like to see some minor changes in how related work is presented, and how the results from fitting the neural network models are presented.

We are very thankful to the reviewers for their extremely helpful comments and suggestions, and their generally positive assessment of our manuscript. We have carefully addressed all comments and detail them point-by-point below. Specifically, we expanded the Introduction and Discussion with additional literature suggested by the reviewer, changed the presentation of Segmentation and Classification performance results in Figs 4 and 5, and made changes to the software as suggested (migrated the GUI to PyQt6, switched to uv, added Sphinx documentation, and added CI testing across three operating systems). We apologize for the issues with installation in the initial version.

For related work, I would strongly suggest revising your description, starting with the paragraph that begins at line 96. While you are of course right about the strengths of your method, it's not quite right to say that "Syllable annotation in neural networks is usually performed in a two-stage process", where you mean "first segmentation, and then classification". You make it sound like this is how *all* neural network models work, which is definitely not the case. The most widely-used neural networks classify each acoustic frame (e.g., spectrogram in a time bin) to *avoid* the need to segment audio and then classify segments. Segments are only recovered by post-processing the frame labels. This is why it is confusing to cite e.g. Steinfath et al. 2021 and then say "classification is only done after the segment onset". You could point out that if you were to rely on such an approach, it would be time consuming to first generate frame-level predictions and then post-process to recover segments; maybe that's what you mean by two-step process? But I think you are referring to the two- step approach of the Schultheiss paper. In principle, there's nothing stopping you from configuring the hyperparameters of DAS or TweetyNet so you have a relatively small network, and then applying it to very small windows. You could even use a similar sliding window approach on the multi-class predictions.

But your key insight as I understand it, drawing inspiration from the Schultheiss paper, is that you can use a much smaller model that is a binary classifier to do online segmentation much faster.

Thank you for this important correction. The original phrasing was too broad and potentially misleading. We have thoroughly rewritten this paragraph to clearly distinguish between (1) framewise classification models such as DAS (Steinfath et al., 2021) and TweetyNet (Cohen et al., 2022), which assign a label to each acoustic frame and recover syllable segments only through post-processing, and (2) approaches that use a dedicated binary segmentation stage followed by a separate classification network (Schulthess et al., 2023; Kawaji et al., 2024). We thank the reviewer for pointing out Pearre et al. (2017), which we had missed in the original manuscript. We now cite it as an early example of neural network-based closed-loop birdsong targeting.

The paragraph in l.101 ff now states:

Several neural network approaches have been developed for closed-loop targeting of birdsong (Pearre et al., 2017), offline annotation (Cohen et al., 2022), or both (Steinfath et al., 2021). Frame classification models (Graves, 2012) such as DAS (Steinfath et al., 2021) and TweetyNet (Cohen et al., 2022) assign a label to each audio frame and recover syllable segments through post-processing. Another approach is to first detect the onsets and offsets of syllable segments ('segmentation') and then classify sound segments into syllable types (Schulthess et al., 2023; Kawaji et al., 2024). Existing tools that follow this two-stage process classify syllable identity only after the segment's offset. However, in many experiments, it is essential to target specific syllable sequences while they are still being vocalized (Tumer and Brainard, 2007; Charlesworth et al., 2011).

I don't want to be pedantic here, and I imagine that you are probably writing for a more impatient reader than me. But I think it is important to not confuse an audience of neuroscientists that may not be as familiar with the AI/ML literature + the details of neural network models. And I think you are missing an opportunity to link what you have done to broader areas of research. There is a couple of important related lines of work that I think you should cite here to put what you're doing in context: (1) AI/ML work on vocal activity detection and (2) bioacoustics/behavioral neuro work that uses lightweight binary classifiers for segmentation.

Following the reviewer's detailed literature suggestions and suggestions of phrasing (see below), we now place Moove's binary segmentation approach within the frameworks of voice activity detection (Hughes and Mierle, 2013) and sound event detection (Stowell, 2022; Kershenbaum et al., 2025), and relate it to work on predicting sequences of animal sounds (Kershenbaum et al., 2016). We cite Baggi et al. (2023) as evidence that lightweight binary classifiers are effective for bioacoustic segmentation; We did not cite the Fukuzawa (2022) thesis as Baggi et al. (2023) covers the same point as a peer-reviewed source. We mention WhisperSeg (Gu et al., 2024) as a transformer-based alternative whose model size makes real-time deployment challenging. Given the word limit of the Introduction, we placed this broader contextualization in the Discussion rather than the Introduction. In the section "Moove can target ongoing syllables", l.526 ff we now state:

Moove's approach can be understood within the broader frameworks of voice activity detection (Hughes and Mierle, 2013) and sound event detection (Stowell, 2022; Kershenbaum et al., 2025), applied to predicting sequences of animal sounds (Kershenbaum et al., 2016). Frame classification models like TweetyNet (Cohen et al., 2022) and DAS (Steinfath et al., 2021) classify each acoustic frame and achieve high offline accuracy through extensive temporal context. TweetyNet uses recurrent layers, which introduce computational overhead and make it primarily designed for offline annotation. With smaller audio chunks, DAS has been used for closed-loop classification of Drosophila song types, but we are not aware of studies using DAS for online birdsong annotation so far. SAIBS (Kawaji et al., 2024) uses threshold-based spectral change detection followed by classification on long temporal windows, which likely include preceding syllables. In the experiment by Kawaji et al., detected syllables were accumulated into a string on which target patterns were matched after bout end, so that feedback is delivered with substantial latency at the bout level rather than during individual syllables. More recently, transformer-based approaches such as WhisperSeg (Gu et al., 2024) have been applied to animal sound segmentation, but are typically orders of magnitude larger, making real-time deployment challenging. Our approach relies on the effectiveness of lightweight binary classifiers for sound segmentation in bioacoustics (Baggi et al., 2023). TinyBird-ML (Schulthess et al., 2023) similarly uses separate networks for real-time detection and classification, but is designed for deployment on wearable sensors with constraints on power and temporal resolution.

Also, both the Schulthess paper and the Kawaji paper provide explicit results from real time experiments, and you are right that both papers report that they classify syllables *after* detection is completed; you should report here the latencies that they report (~4 ms for classification in Schultheiss and ~109 ms for Kawaji) here in the intro as a motivation for your approach! We thank the reviewer for pointing out these values and agree that comparisons based on concrete latency values from related work would be valuable. However, after carefully examining both papers, we concluded that citing these figures as benchmarks would be misleading rather than informative.

For Kawaji et al. (SAIBS), classification occurs after syllable offset on a 232 ms spectrogram window (40 frames of 5.8 ms) flattened to a 1D vector and passed to MATLAB's patternnet, a fully-connected feedforward network, rather than the CNN described in the paper. Target detection operates by substring matching on the accumulated sequence string, and the reported 109 ms latency refers to a bout-level reward trigger, gated by a 2 s silence timeout. Neither quantity maps onto the syllable-onset-based latency that we report for Moove. Further, annotation accuracy in SAIBS is reported against TweetyNet, which was itself trained on SAIBS- generated labels, so the comparison reflects self-consistency rather than accuracy against an independent ground truth.

For Schulthess et al. (TinyBird-ML), the 4 ms figure reflects on-chip inference time only, not end-to-end closed-loop latency. Classification occurs after syllable offset and the system operates at 16 kHz versus our 44.1 kHz, so the effective latency is at minimum one full syllable duration plus offset-detection delay.

Citing either figure without these caveats would misrepresent both works and our own. We therefore opted against direct numerical comparison in the main text, while addressing the qualitative differences in the Discussion section (see paragraph quoted above).

I'll just say how I would describe things, and you are of course free to integrate any of these suggestions however you choose. Here's my version (apologies for long-windedness, I get this is not everyone's style).

For the method we develop here, there are two ways that neural network models have been applied to laboratory studies of animal acoustic communication that are most relevant. The first way consists of fitting relatively large models that can be used to segment and classify animal sounds in an off-line manner, or potentially on-line, as we propose. These are typically frame classification models (Graves 2012) like DAS (Steinfath 2021) or TweetyNet (Cohen 2022) that classify each acoustic frame (e.g., a timebin in a spectrogram, or in some cases audio samples). Classified segments can only be recovered from frame-level predictions by post-processing. These models can be seen as sound event detection models (Stowell 2022, Bioacoustics review) or voice activity detection models (e.g., Hughes Mierle 2013) applied specifically to recordings of sounds made by single acoustically-isolated individual animals. In other words, the models are used to predict sequences of sound (Kershenbaum et al 2016), not just to detect the presence or absence of sound (Kershenbaum et al 2025). More recently, a method to segment animal sounds using transformer models (cite WhisperSeg) has been proposed. However, transformers like those used as the backbones of Large Language Models are typically orders of magnitude larger than the frame classification models of Steinfath 2021 and Cohen 2022, and so would be challenging to use in a setting like ours, although there are ways to accelerate inference. The Temporal Convolutional-based architecture of Steinfath et al 2021, in principle, enables faster inference than the recurrent architecture of Cohen et al 2022, and this is supported by their benchmarking results. (Aside: Steinfath et al 2021 repeatedly claims their architecture enables closed-loop experiments but I do not believe there are any results from actual closed-loop experiments in the paper, and I was not able to find any citing work that does such experiments -- if published results exist, you should cite them here or somewhere else, and you should include any measures of latency from closed-loop experiments with DAS, especially if those papers include any information on factors like the inference window size).

As stated above, we incorporated this broader conceptual framing and the suggested literature in the revised Discussion section. Regarding the closed-loop capability of DAS, we confirmed the reviewer's impression that there seem to be no published applications of DAS in closed-loop experiments, and therefore reference the latency tests with Drosophila song types in the original Steinfath et al. 2021 paper.

The second way neural network models have been used directly aligns with our approach here: to target acoustic communication for closed-loop experimental interventions. Neural networks have been used to target birdsong at least as early as Pearre et al 2017: https://journals.plos.org/plosone/article?id=10.1371/journal.pone.018199. (Aside: AFAICT you don't cite this paper but I think it's the most directly related to your work, except for the Schultheiss paper? Again you should include any relevant measures of inference latency, etc.). More recent work from Kawaji and Schultheiss is most relevant for our approach here. The approach of Kawaji reports 109 ms latency for targeting, while the approach of Schultheiss et al reports a latency of 4 ms. (Aside: I find the Kawaji paper hard to grok but if possible you should give a short summary of their approach, to explain why the inference time is so high compared to yours.) We draw direct inspiration from the approach of Schultheiss, using a two-stage model that begins with binary classification of each frame as either syllable or background, and when enough frames of a syllable are detected, we then classify the syllable with a separate network. Their key insight that we build on is that a binary segmentation model can be much smaller and faster, allowing for the faster inference needed for our syllable targeting. The strength of this approach is also by previous work using quite small neural networks for binary segmentation (Fukuzawa thesis, Baggi et al 2023). Crucially, we modify the Schultheiss approach so that we can classify a syllable after detecting an onset, the start of a syllable, instead of waiting until after we detect an offset, the end of a syllable. As we show, this allows us to reduce detection times down to 1 ms Papers I'm citing that you might not know:

Graves, Alex. "Supervised sequence labelling." Supervised sequence labelling with recurrent neural networks. Berlin, Heidelberg: Springer Berlin Heidelberg, 2012. 5-13.

Graves, Alex, and Jürgen Schmidhuber. "Framewise phoneme classification with bidirectional LSTM and other neural network architectures." Neural networks 18.5-6 (2005): 602-610.

Stowell, Dan. "Computational bioacoustics with deep learning: a review and roadmap." PeerJ 10 (2022): e13152. Hughes, Thad, and Keir Mierle. "Recurrent neural networks for voice activity detection." 2013 IEEE International Conference on Acoustics, Speech and Signal Processing. IEEE, 2013.

Kershenbaum, Arik, et al. "Acoustic sequences in non‐human animals: a tutorial review and prospectus." Biological Reviews 91.1 (2016): 13-52.

Kershenbaum, Arik, et al. "Automatic detection for bioacoustic research: a practical guide from and for biologists and computer scientists." Biological Reviews 100.2 (2025): 620-646.

Fukuzawa, Yukio. Computational methods for a generalised acoustics analysis workflow: A thesis presented in partial fulfilment of the requirements for the degree of Master of Science in Computer Science at Massey University, Auckland, New Zealand. Diss. Massey University, 2022.

Baggi et al. 2023: https://www.nature.com/articles/s41598-023-38186-7 As stated above, we incorporated these suggestions into the revised Introduction and Discussion sections. As a brief summary of SAIBS's approach, to carify the architectural differences from Moove:

SAIBS (Kawaji et al., 2024) uses threshold-based spectral change detection followed by classification on long temporal windows, which likely include preceding syllables. In the experiment by Kawaji et al., detected syllables were accumulated into a string on which target patterns were matched after bout end, so that feedback is delivered with substantial latency at the bout level rather than during individual syllables.

For the results from fitting neural networks, here's what I would do a bit differently. While figures 4 and 5 are helpful, in that they show that there is no huge difference between training accuracy and test accuracy, besides that I find them a bit hard to read, and the differences in accuracy leave me wondering what might account for them. For me, tables 1 and 2 are much more informative than figures 4 and 5, but I would really like to see you quantify things a bit more to help readers understand the ins and outs of your approach. For table 1 (and figure 4): accuracy is not a super-informative metric when fitting binary classification models. You should check but IME you typically are in an unbalanced setting where there are far more frames of background class than foreground class, and so it's very easy to get a "high accuracy" simply by predicting 0 for every frame. This is a failure mode that neural networks can go into when you are fitting with binary cross entropy. Although your results clearly show that's not happening here, it's something to be aware of; you don't say if you do this in the methods, but you could weight the BCE loss to mitigate this effect. For the binary classifier, you probably want to compute framewise precision, recall, and F1, like in the Steinfath 2021 paper. I see in the text you give P/R/F1 scores for bird 1 only, but you don't state in the methods how you compute these metrics; I'm guessing they are in fact the framewise metrics like in the Steinfath 2021 paper? Here's what I would really like to see in table 1: Training set size in seconds (I would prefer this over number of bouts, since bout length can vary both by bout and by bird); if you think it's necessary, training and test acc/err; ideally, framewise P/R/F1, and additionally the same information-retrieval style metrics, but computed for segmentation, like those here: https://vocalpy.readthedocs.io/en/latest/api/generated/vocalpy.metrics.segmentation.fscore.html#vocalpy. metrics.segmentation. Please make it very clear that these are *segmentation* metrics, and not e.g. framewise precision/recall/F1, and also please make it very clear what tolerance/collar value you use (e.g., 10 ms, although note that is probably a little too generous for what you care about. You might present these scores computed with multiple collar values as is coming, e.g. $P_{seg}@10$, $P_{seg}@5$, where the number indicates the collar size in milliseconds). You should also include the same segmentation metrics for the energy-based segmentation method, to show your model does better than that approach. I also think it would be really good to run e.g. three training replicates for each bird, and then include mean/standard deviation of all the metrics. This will better support your claim that your approach to fitting the networks. That would probably be more informative than giving the final test loss. I am not sure you get a lot out of reporting specific loss values. For figure 4, instead of just plotting what you already have in tables, what I would really like to see is some sort of analysis that helps us understand the differences in metrics. For example, if you plot training set size in seconds vs test accuracy (or segmentation P/R/F1) is there a correlation? By eyeballing table 4, I think you would. It would be good to know how training set size impacts performance. For the loss plots, instead of showing final loss values, it would probably be more helpful if you log the loss scores during training and then show plots of loss across training steps, to support your claims that the optimization is generally well-behaved and converges smoothly; the least painful way to do this will probably be to use the Summarywriter logger built into Pytorch - - see here for how to get the logged data out into a format you can plot: https://github.com/vocalpy/vak/blob/main/src/vak/common/tensorboard.py. You can plot e.g. all the training histories from individual runs, and then a mean across runs as a darker line.

We thank the reviewer for these detailed suggestions. We have thoroughly revised the results section to include all proposed analyses. Figure 4B now shows framewise precision, recall, and F1 for all five birds. Table 1 has been changed accordingly, and now includes training set size in seconds. We have now run three training replicates per bird for both networks, with all metrics reported as mean {plus minus} SD. Figure 4A now shows loss curves over training epochs. Figure 4D shows training set duration versus framewise F1, confirming the positive association suspected by the reviewer. Figure 4C shows segment-level onset-based F1 scores with collar tolerances of 5, 10, 15, and 20 ms (see also Table 1). We have taken care to clearly distinguish these from framewise metrics. The same segmentation metrics are reported for the energy-based baseline, showing that Moove outperforms it for four of five birds.

We also thank the reviewer for pointing out the importance of class imbalance in binary frame-level segmentation. We have updated the training procedure so that the segmentation network now uses positive class weighting in the binary cross-entropy loss by default. Downsampling remains available as an option, but class weighting is now the default approach. The classification network uses inverse-frequency class weights to account for class imbalance between syllable classes. These changes have been implemented in the code and clarified in the Methods. We did not remove accuracy entirely from the classification table (Table 2) as it remains informative for multi-class classification. Table 3 additionally quantifies the trade-off between input duration and classification accuracy across birds, supporting the choice of 30 ms as the default window.

Minor comments and suggestions:

- 141 - "but require faster inference" is confusingly worded -- maybe you mean "reduce latency and allow for faster inference"? The phrasing has been updated as suggested.

- 148 -- AI/ML folks would probably refer to this as detection, worth explaining the link between "segmentation" and "binary classification" here in the intro instead of deferring to methods/results (if you didn't already do this in a revised related work section) The link between segmentation and binary frame-level classification is now made explicit in the Methods section, as follows. Because of word limitations, we could not go into such detail in the Introduction section:

The segmentation network is a dedicated network for the detection of syllable segments in a continuous stream of audio data. It operates on very short chunks to perform binary frame-level classification of presence or absence of syllables. A second network (the classification network) classifies the detected syllable segments into syllable types.

- 155 - "to determine segment membership (syllable/gap)." -- I would say this as "...(MLP) that we fit as a binary classifier, to classify each frame as belonging to either our class of interest, syllables, or the background class, consisting of silent gaps between syllables and all other non-syllable sounds" We have adopted the reviewer's suggested phrasing, now describing the MLP as classifying "each frame as belonging to either a syllable class or the background class, consisting of silent gaps and all other non-syllable sounds." - 155 - minor point about phrasing: I would phrase this as "Using the default parameters of a chunk size of 64 samples at 44.1 kHz, this network makes approximately 690 inferences per second." Rephrased as suggested - 236 -- it's a bit confusing to say "algorithm-driven segmentation" here; a neural network is an algorithm! Especially once you add post-processing and a whole data pipeline around. I know the songbird neuro literature often uses the term "amplitude thresholding", that I think might be inherited from SAP, but engineers and bioacoustics folks will probably be more familiar with a term like "energy-based segmentation methods", see https://ieeexplore.ieee.org/abstract/document/861862 (as cited e.g. on https://vocalpy.readthedocs.io/en/latest/api/generated/vocalpy.metrics.segmentation.fscore.html#vocalpy.me trics.segmentation.fscore) and https://besjournals.onlinelibrary.wiley.com/doi/full/10.1111/2041-210X.14170 -- adding this would help you distinguish between a purely signal processing-based method, and your method that segments by applying a sliding window to framewise binary neural net outputs (which may also be prone to oversegmentation! which will manifest itself as a high recall score for segmentation) We have replaced "algorithm-driven segmentation" and "amplitude thresholding" with "energy-based segmentation" throughout the manuscript. We agree this more clearly distinguishes the signal processing-based baseline from Moove's approach.

- 249 "with logits" is an implementation detail, just say "binary cross entropy" Changed accordingly throughout - For Fig. 7, where the authors target the 4th syllable in a repeat phrase, it is unclear how reliably they were able to do this. Some accuracy measures along with false positive and false negative proportions We apologize. Fig 7 was just meant to illustrate one potential use of Moove to target repeat phrases, and this experiment will be featured more quantitatively in an upcoming publication. We have now 1) changed phrasing in this section to clarify that this figure does not represent a quantitative test of classification performance: "As an illustration of this use case, we show an example song bout in which we targeted the fifth repetition of syllable 'a' within a repeat phrase for auditory feedback manipulations" 2) As suggested, we have also added quantitative targeting performance for this particular experiment to the section: "Within this example experiment, Moove correctly triggered on 34 of 36 target phrases, with one false positive (precision:

97.1%, recall: 94.4%, F1 = 0.96), demonstrating its usefulness for targeting specific positions within repeat phrases." - Line 81, I feel that you could use "negative reinforcement" to further emphasize the role of WN in this paradigm We appreciate the suggestion. However, in strict behavioral psychology terminology, the WN paradigm could be interpreted as constituting a positive punishment (an aversive stimulus is added, leading to reduction of the targeted behavior) rather than negative reinforcement (removal of an aversive stimulus to increase behavior). We have added "aversive" to emphasize the role of WN, while remaining agnostic whether the bird learns from punished or non-punished renditions, or both.

- Line 199 - other feedback modalities can be discussed a little more in the Discussion along with possible ways to implement these.

The Discussion now includes a slightly expanded description of potential feedback modalities, noting that adapting Moove would primarily require modifying the feedback output module, as the segmentation and classification pipeline is modality-independent:

Moove's real-time targeting capability supports diverse experimental applications beyond the operant conditioning protocol presented here. The system could be used for song-triggered manipulations of auditory feedback (Tumer and Brainard, 2007), visual feedback (Zai et al., 2020), somatosensory feedback (McGregor et al., 2022), neural microstimulation (Moll et al., 2023), optogenetic perturbations (Hisey et al., 2018), and access to conspecifics (Ben-Tov et al., 2023). Adapting Moove for these modalities would primarily require modifying the feedback output module, as the segmentation and classification pipeline is modality-independent.

- Line 250 - what are the other hyperparameters that were set for all of the neural network models (learning rate, etc.) All hyperparameters are now explicitly stated in the Methods for both networks, including optimizer (Adam), learning rate (0.001), batch size (64), early stopping patience (5 epochs), dropout rates, and data split ratios.

- Line 277 - please explain what the different data augmentation techniques do and which ones were actually used in the code All four augmentation techniques are now described with their parameters in the Methods: additive Gaussian noise (0.0001), frequency masking (up to 10 bins), time masking (up to 10 frames), and dynamic range compression (factor 0.5). Each augmented sample receives one randomly selected technique. No data augmentation was used for training the segmentation network.

- Line 320, 323 - specify whether the errors are SD or SEM. Providing the median would also be helpful as the mean is influenced by outliers) All error measures are now specified as SD throughout. Median values are provided where relevant, including for onset timing errors (median: −1.5 ms).

- Line 346 - another measure of how well segmentation was done would be to provide statistics for the difference between automatically segmented onsets/offsets and manually segmented onsets/offsets We now report the difference between automatically detected and manually annotated onsets across all birds and replicates (n = 24,277 matched onsets): mean onset error was −0.5 {plus minus} 1.9 ms (median: −1.5 ms), confirming that detected onsets closely match annotated boundaries. We did not report offset error, as onset timing is the relevant measure for closed-loop targeting where feedback is triggered relative to syllable onset.

- Fig. 6 - it is unclear which repeat was targeted? Was the 2nd 'a' targeted? Clarify The figure caption now specifies that the second occurrence of syllable 'a' within the repeat phrase was targeted with white noise playback.

- Line 465 - unclear what is the original definition of catch trials and what it has been changed into. Clarify.

- Line 472-477 - it is not clear how transition probability is calculated and how the resampling procedure was done. Was transition probability only calculated on catch trial bouts? We have clarified that in this experiment catch trials were mistakenly assigned only to bouts containing the target sequence, and that this differs from the standard definition where catch trials are randomly sampled from all bouts regardless of content. The implementation has since been updated in Moove to use the standard definition of catch trials in the field. We have added a detailed description of how transition probabilities are calculated and how the resampling procedure corrects for the sampling bias introduced by the non-standard catch trial assignment. We have moved this more detailed explanation into the Methods section:

In this earlier version of Moove, catch trials were mistakenly only assigned to bouts that contained the target sequence. Therefore, transition probabilities computed from catch-trial bouts alone may overestimate the occurrence of the target transition. To correct for this sampling bias, we performed a resampling procedure: we randomly sampled 10% of all bouts and replaced any non -catch-trial bouts containing the target sequence with randomly drawn catch-trial bouts (with replacement); any non- catch-trial bouts that did not contain the target sequence also did not contain WN, and could therefore be treated like catch trials. We repeated this resampling for 1000 iterations and from the resulting distribution, we extracted minimum, median, and maximum transition probabilities. This simulates the effect that the proper assignment of catch trials to all bouts would have had on the analysis.

Transition probabilities were calculated as the proportion of transitions from 'h-c' to each following syllable across all bouts during baseline, and post-screening periods, and in resampled catch trial bouts during the training period.

- Line 487 - When were the post-screening sessions conducted? How many days after training stopped? Post-screening sessions were conducted 16 and 37 days after the last training day, respectively. This information has been added to the Methods.

Specific comments on the software:

The tool described in the paper is a new software. I was able to download it and install it. The GUI initiated but there were issues with versions of different python libraries. I suspect this has more to do with my laptop having an older version of linux. Therefore, I cannot comment on testing of the software.

We thank the reviewer for pointing out this issue. The migration from Tkinter to PyQt6 and from Poetry to uv/hatchling (see below) should resolve the library version conflicts encountered on Linux.

I would really like to have installed and tested the package but I ran into multiple issues. I do not think this is the authors' fault, it is a simple reflection of the state of the scientific Python ecosystem, particularly around working with sound at a low-level and with developing GUIs. I am providing more concrete feedback here but I do not think any of this should hold up review, rather issues should be raised on the GitHub repository and considered for future development of the package. I reiterate that I am confident the software works as advertised, and that the authors are surely willing to help other labs get it up and running; my comments here are just advice to help them make that easier in the long run.

We appreciate the reviewer's understanding. We have substantially improved the installation experience: the build system has been migrated from Poetry to uv/hatchling, the GUI from Tkinter to PyQt6, and GitHub Actions CI now runs our test suite on Linux, macOS, and Windows to catch platform-specific issues early.

My initial review of the GitHub repository suggests that it meets most criteria for "good enough" scientific software, as discussed in https://journals.plos.org/ploscompbiol/article?id=10.1371/journal.pcbi.1005510#sec027. I notice that there are no unit tests and no separate docs. I completely understand that it's more pragmatic to write the docs in something like word and then posting a PDF to the repo, but long term you probably waI would really like to have installed and tested the package but I ran into multiple issues. I do not think this is the authors' fault, it is a simple reflection of the state of the scientific Python ecosystem, particularly around working with sound at a low-level and with developing GUIs. I am providing more concrete feedback here but I do not think any of this should hold up review, rather issues should be raised on the GitHub repository and considered for future development of the package. I reiterate that I am confident the software works as advertised, and that the authors are surely willing to help other labs get it up and running; my comments here are just advice to help them make that easier in the long run.

My initial review of the GitHub repository suggests that it meets most criteria for "good enough" scientific software, as discussed in https://journals.plos.org/ploscompbiol/article?id=10.1371/journal.pcbi.1005510#sec027. I notice that there are no unit tests and no separate docs. I completely understand that it's more pragmatic to write the docs in something like word and then posting a PDF to the repo, but long term you probably want to have a separately rendered docs site. For writing unit tests and setting up a docs site, I'd suggest consulting, the following:

- https://www.pyopensci.org/python-package-guide/ - https://learn.scientific-python.org/development/ We followed the guidance from both resources when setting up the test suite and documentation infrastructure. A comprehensive test suite with 205 passing tests has been added, organized across unit, component, and end-to-end levels. A Sphinx documentation site has been set up, converting the existing Word- based guide into eight structured chapters. Both the tests and documentation build are integrated into the CI pipeline.

Again, the following is provided as feedback, none of these should hold up the review.

I was not able to install on Linux because of the dependency on tkinter -- there is just no easy way to set up a separate install of python built up with tkinter, especially if you already have multiple versions of python installed with something like pyenv, and you don't want to break any existing installs. I then switched to Windows but here I ran into issues because of poetry. Even after upgrading my poetry version I still got errors from `poetry install` complaining that "name", "version", etc. are missing but required in package mode. I think this cryptic error is because I have an install of `poetry` with `pipx` and it's running in some location other than the moove repo, as described here:https://github.com/python-poetry/poetry/issues/10029.

The GUI has been fully migrated from Tkinter to PyQt6, eliminating the tkinter dependency entirely. The build system has been migrated from Poetry to uv/hatchling, resolving the "missing name/version in package mode" errors encountered with pipx-installed Poetry.

If you want to make the package more widely accessible I would suggest using PyQt as your GUI framework to avoid the issues of tkinter, Qt has the most Python support. You will want to use either VisPy to render plots, like napari does, or you will need to use PyQTGraph like the DAS GUI, but for my taste the latter is over- engineered.

We have migrated the entire GUI to PyQt6 as suggested. We appreciate the pointer to VisPy and PyQtGraph for plot rendering and will consider these for future development.

I would also suggest using uv over poetry as your Swiss-army-knife development tool -- poetry is designed largely by and for people deploying web apps, and so they make some design choices that are not great for scientific Python developers, as discussed at length here: https://iscinumpy.dev/post/bound-version- constraints/. I think this has improved somewhat in recent versions of poetry, but I'm guessing you will find life easier in the long run if you switch to uv and/or pixi, although you will have to pay the cost of switching in the short term.

We have switched from Poetry to uv as suggested, with dev-dependencies now using PEP 735 dependency groups and the poetry.lock replaced by uv.lock for reproducible installs.

Likewise there are things you can do to mitigate the headaches caused by the dependency on sounddevice -- please know I am not saying this is your fault; sounddevice is the least bad option, but as your installation docs make clear, there's a lot of platform-specific set-up required. I bet that if you make a conda-forge package for moove you can avoid some of the pain of installing a system-wide portaudio package. There are conda-forge packages for both portaudio and sounddevice (conda-forge requires all your dependencies have conda-forge pacakges themselves). Conda-forge recipes can be surprisingly painless to set up, using greyskull, see: https://conda-forge.org/docs/maintainer/adding_pkgs/#generating-the-recipe We thank the reviewer for this helpful suggestion. We have prepared a conda-forge recipe for Moove and submitted a pull request to the conda-forge staged-recipes repository. However, the PortAudio package available on conda-forge is currently built without ASIO support. Users requiring low-latency ASIO audio on Windows should therefore follow the pip-based installation procedure described in the documentation.

If these were issues for me, someone who supposedly knows what they are doing, then I'm guessing other people with less experience will run into this problems and in many cases just give up. I would encourage you to invest the development and maintenance time needed to provide alternatives.

We appreciate this perspective and have prioritized low-barrier installation paths. We migrated the GUI to PyQt6, switched to uv, added Sphinx documentation with a troubleshooting section, prepared a conda-forge recipe pending release, and added CI testing across three operating systems to substantially lower the barrier for new users.

One other minor detail I noticed from the README and docs: it is surprising to see required versions specified as less than a certain number (e.g., torch < 2.6). Typically for library development you want a lower bound on versions. See https://snarky.ca/why-i-dont-like-semver/ (or for a very in-depth explanation, again see: https://iscinumpy.dev/post/bound-version-constraints/). I know this rule of thumb about dependencies isn't obvious (see here http://mrussek.com/2024/01/02/python-library-dependencies.html) but IME lower-bounds with no upper caps is the least painful to deal with -- most core libraries in the scientific Python stack that you depend on will try to adhere to SemVer, with the implicit guarantee that they won't make any breaking changes to the API without a major increment in the version number.

All upper version bounds have been removed from dependency constraints, following the reviewer's recommendation and best practices for scientific Python packages.
