# Supplementary figures and images for: Real-Time Segmentation and Classification of Birdsong Syllables for Learning Experiments
Source: eNeuro. 2026 Jun 30;13(7):ENEURO.0023-26.2026. doi: 10.1523/ENEURO.0023-26.2026 (PMC13326709; doi:10.1523/ENEURO.0023-26.2026)

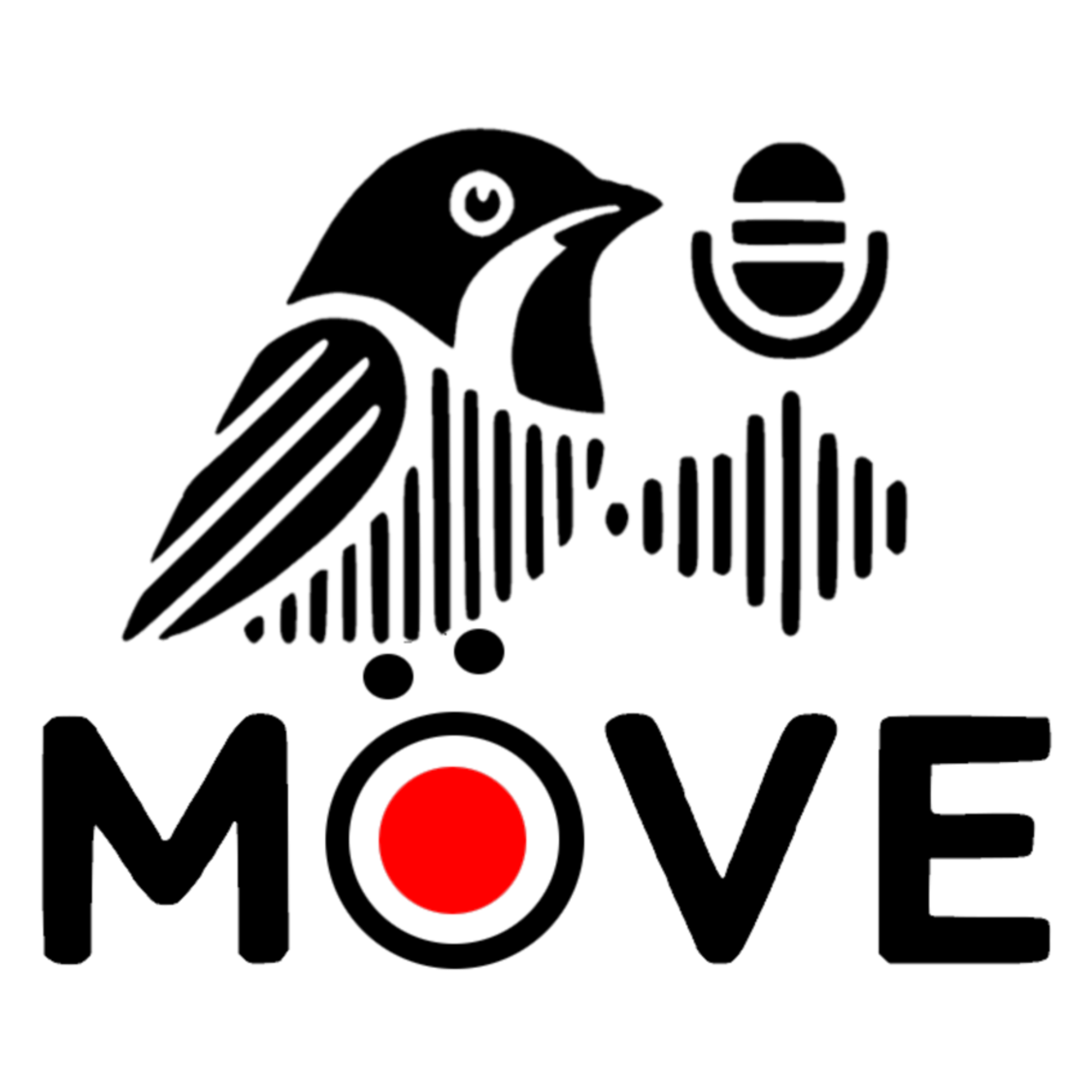

Supplement: Data 1 — The complete Python source code for MooveTAF (real-time recording and targeting) and MooveGUI (data preprocessing, labeling, and network training), packaged as a single ZIP archive. The latest version is available at: https://github.com/veitlab/moove Download Data 1, ZIP file. [file eneuro-13-ENEURO.0023-26.2026-s001.zip › moove-main/assets/logo.png]

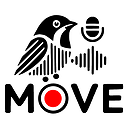

Supplement: Data 1 — The complete Python source code for MooveTAF (real-time recording and targeting) and MooveGUI (data preprocessing, labeling, and network training), packaged as a single ZIP archive. The latest version is available at: https://github.com/veitlab/moove Download Data 1, ZIP file. [file eneuro-13-ENEURO.0023-26.2026-s001.zip › moove-main/assets/logo_128.png]

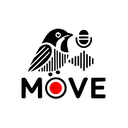

Supplement: Data 1 — The complete Python source code for MooveTAF (real-time recording and targeting) and MooveGUI (data preprocessing, labeling, and network training), packaged as a single ZIP archive. The latest version is available at: https://github.com/veitlab/moove Download Data 1, ZIP file. [file eneuro-13-ENEURO.0023-26.2026-s001.zip › moove-main/assets/logo_128_white_bg.png]

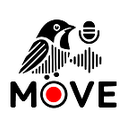

Supplement: Data 1 — The complete Python source code for MooveTAF (real-time recording and targeting) and MooveGUI (data preprocessing, labeling, and network training), packaged as a single ZIP archive. The latest version is available at: https://github.com/veitlab/moove Download Data 1, ZIP file. [file eneuro-13-ENEURO.0023-26.2026-s001.zip › moove-main/assets/logo_128_white_bg_scaled.png]

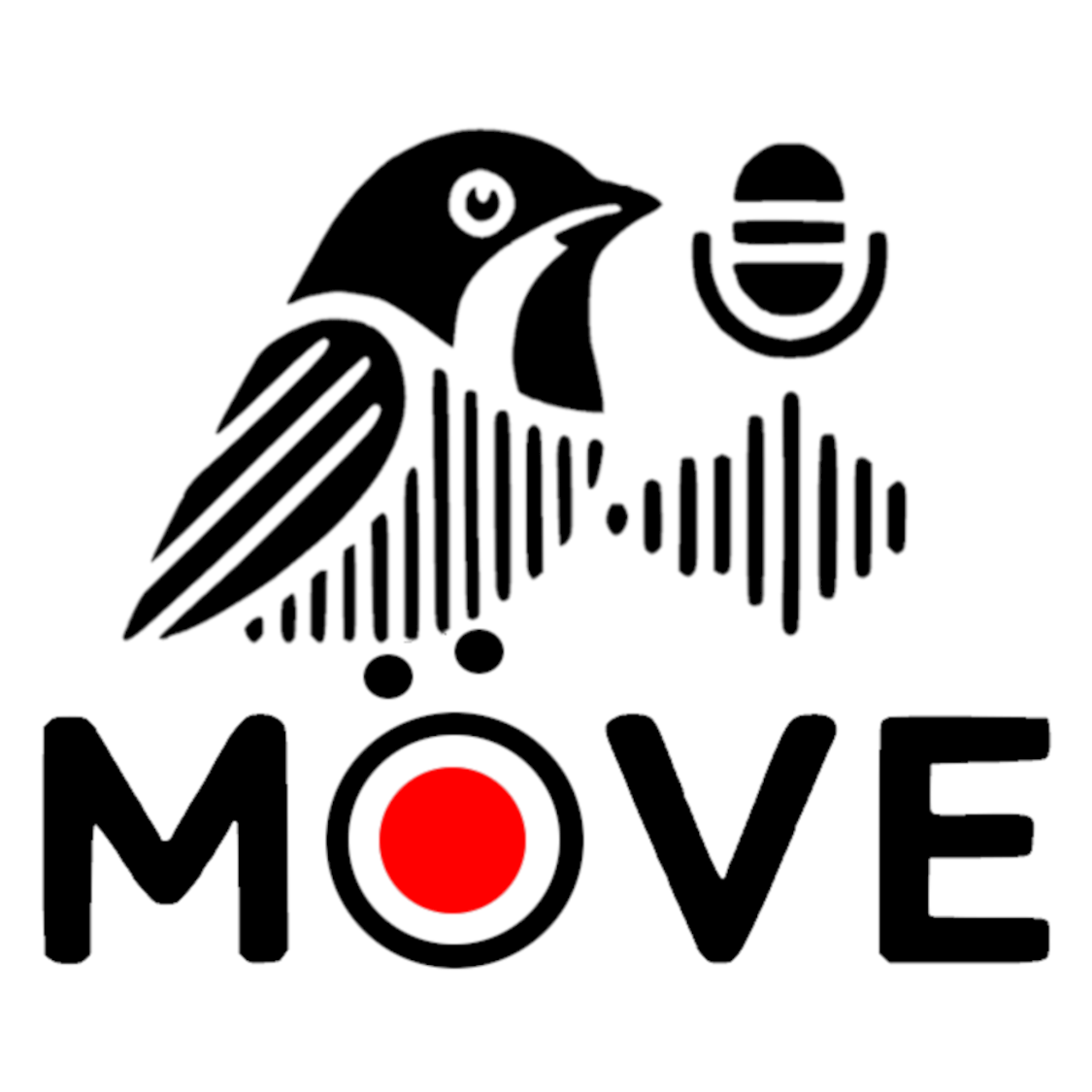

Supplement: Data 1 — The complete Python source code for MooveTAF (real-time recording and targeting) and MooveGUI (data preprocessing, labeling, and network training), packaged as a single ZIP archive. The latest version is available at: https://github.com/veitlab/moove Download Data 1, ZIP file. [file eneuro-13-ENEURO.0023-26.2026-s001.zip › moove-main/assets/logo_white_bg.png]

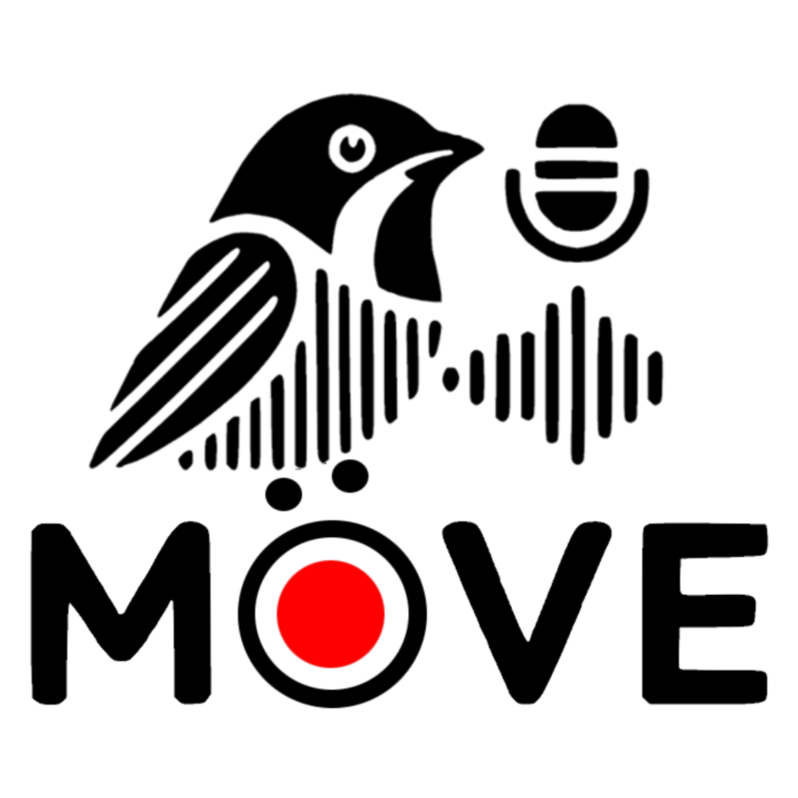

Supplement: Data 1 — The complete Python source code for MooveTAF (real-time recording and targeting) and MooveGUI (data preprocessing, labeling, and network training), packaged as a single ZIP archive. The latest version is available at: https://github.com/veitlab/moove Download Data 1, ZIP file. [file eneuro-13-ENEURO.0023-26.2026-s001.zip › moove-main/docs/source/_static/images/image1.png]

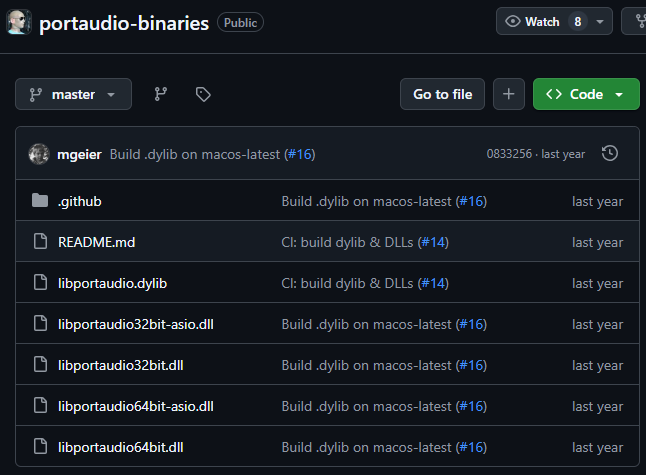

Supplement: Data 1 — The complete Python source code for MooveTAF (real-time recording and targeting) and MooveGUI (data preprocessing, labeling, and network training), packaged as a single ZIP archive. The latest version is available at: https://github.com/veitlab/moove Download Data 1, ZIP file. [file eneuro-13-ENEURO.0023-26.2026-s001.zip › moove-main/docs/source/_static/images/image10.png]

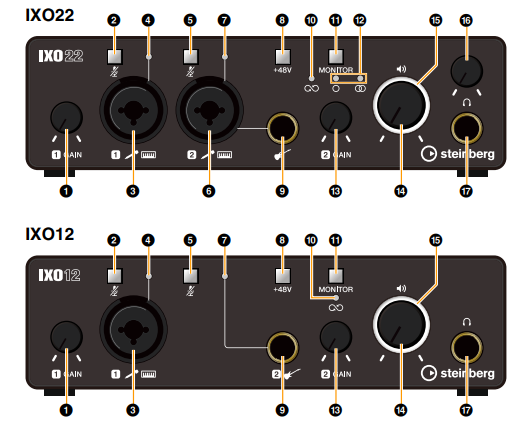

Supplement: Data 1 — The complete Python source code for MooveTAF (real-time recording and targeting) and MooveGUI (data preprocessing, labeling, and network training), packaged as a single ZIP archive. The latest version is available at: https://github.com/veitlab/moove Download Data 1, ZIP file. [file eneuro-13-ENEURO.0023-26.2026-s001.zip › moove-main/docs/source/_static/images/image11.png]

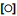

Supplement: Data 1 — The complete Python source code for MooveTAF (real-time recording and targeting) and MooveGUI (data preprocessing, labeling, and network training), packaged as a single ZIP archive. The latest version is available at: https://github.com/veitlab/moove Download Data 1, ZIP file. [file eneuro-13-ENEURO.0023-26.2026-s001.zip › moove-main/docs/source/_static/images/image12.png]

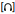

Supplement: Data 1 — The complete Python source code for MooveTAF (real-time recording and targeting) and MooveGUI (data preprocessing, labeling, and network training), packaged as a single ZIP archive. The latest version is available at: https://github.com/veitlab/moove Download Data 1, ZIP file. [file eneuro-13-ENEURO.0023-26.2026-s001.zip › moove-main/docs/source/_static/images/image13.png]

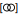

Supplement: Data 1 — The complete Python source code for MooveTAF (real-time recording and targeting) and MooveGUI (data preprocessing, labeling, and network training), packaged as a single ZIP archive. The latest version is available at: https://github.com/veitlab/moove Download Data 1, ZIP file. [file eneuro-13-ENEURO.0023-26.2026-s001.zip › moove-main/docs/source/_static/images/image14.png]

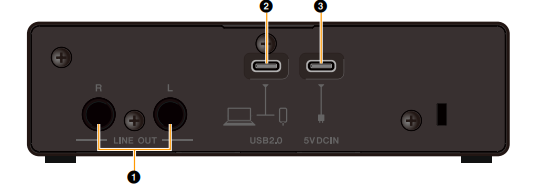

Supplement: Data 1 — The complete Python source code for MooveTAF (real-time recording and targeting) and MooveGUI (data preprocessing, labeling, and network training), packaged as a single ZIP archive. The latest version is available at: https://github.com/veitlab/moove Download Data 1, ZIP file. [file eneuro-13-ENEURO.0023-26.2026-s001.zip › moove-main/docs/source/_static/images/image15.png]

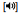

Supplement: Data 1 — The complete Python source code for MooveTAF (real-time recording and targeting) and MooveGUI (data preprocessing, labeling, and network training), packaged as a single ZIP archive. The latest version is available at: https://github.com/veitlab/moove Download Data 1, ZIP file. [file eneuro-13-ENEURO.0023-26.2026-s001.zip › moove-main/docs/source/_static/images/image16.png]

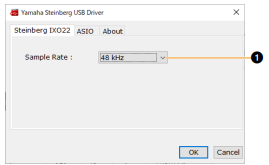

Supplement: Data 1 — The complete Python source code for MooveTAF (real-time recording and targeting) and MooveGUI (data preprocessing, labeling, and network training), packaged as a single ZIP archive. The latest version is available at: https://github.com/veitlab/moove Download Data 1, ZIP file. [file eneuro-13-ENEURO.0023-26.2026-s001.zip › moove-main/docs/source/_static/images/image17.png]

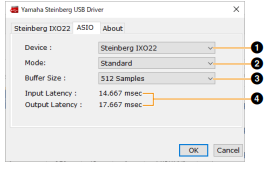

Supplement: Data 1 — The complete Python source code for MooveTAF (real-time recording and targeting) and MooveGUI (data preprocessing, labeling, and network training), packaged as a single ZIP archive. The latest version is available at: https://github.com/veitlab/moove Download Data 1, ZIP file. [file eneuro-13-ENEURO.0023-26.2026-s001.zip › moove-main/docs/source/_static/images/image18.png]

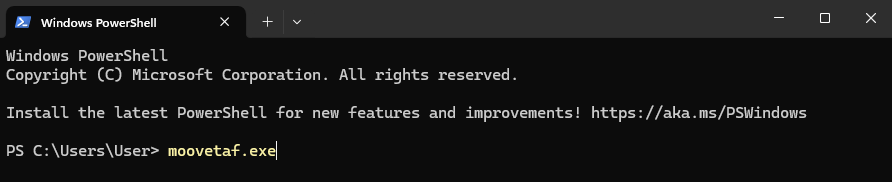

Supplement: Data 1 — The complete Python source code for MooveTAF (real-time recording and targeting) and MooveGUI (data preprocessing, labeling, and network training), packaged as a single ZIP archive. The latest version is available at: https://github.com/veitlab/moove Download Data 1, ZIP file. [file eneuro-13-ENEURO.0023-26.2026-s001.zip › moove-main/docs/source/_static/images/image19.png]

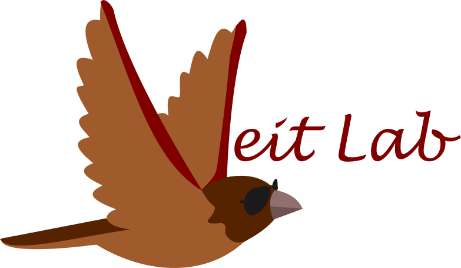

Supplement: Data 1 — The complete Python source code for MooveTAF (real-time recording and targeting) and MooveGUI (data preprocessing, labeling, and network training), packaged as a single ZIP archive. The latest version is available at: https://github.com/veitlab/moove Download Data 1, ZIP file. [file eneuro-13-ENEURO.0023-26.2026-s001.zip › moove-main/docs/source/_static/images/image2.png]

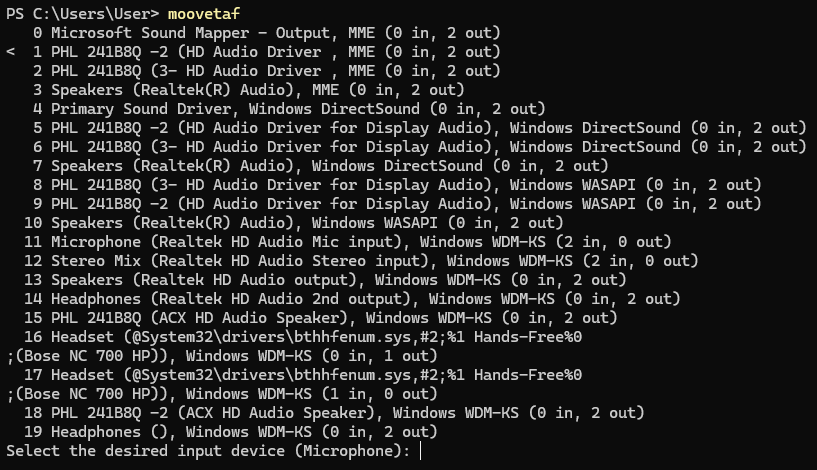

Supplement: Data 1 — The complete Python source code for MooveTAF (real-time recording and targeting) and MooveGUI (data preprocessing, labeling, and network training), packaged as a single ZIP archive. The latest version is available at: https://github.com/veitlab/moove Download Data 1, ZIP file. [file eneuro-13-ENEURO.0023-26.2026-s001.zip › moove-main/docs/source/_static/images/image20.png]

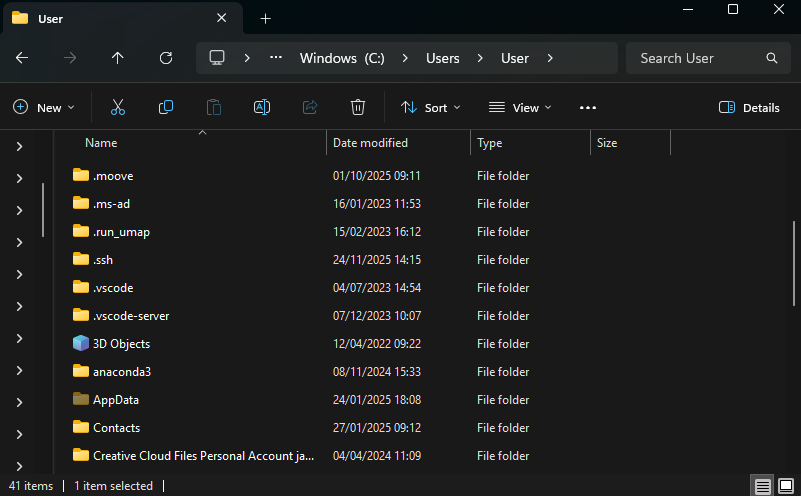

Supplement: Data 1 — The complete Python source code for MooveTAF (real-time recording and targeting) and MooveGUI (data preprocessing, labeling, and network training), packaged as a single ZIP archive. The latest version is available at: https://github.com/veitlab/moove Download Data 1, ZIP file. [file eneuro-13-ENEURO.0023-26.2026-s001.zip › moove-main/docs/source/_static/images/image21.png]

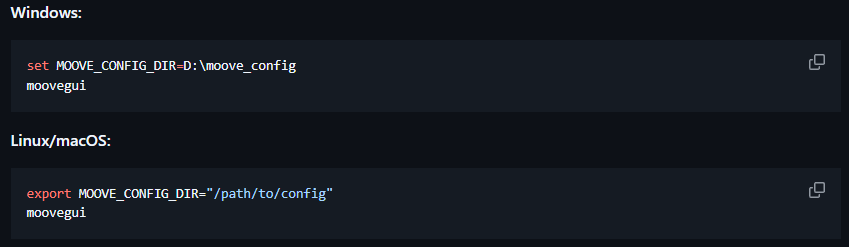

Supplement: Data 1 — The complete Python source code for MooveTAF (real-time recording and targeting) and MooveGUI (data preprocessing, labeling, and network training), packaged as a single ZIP archive. The latest version is available at: https://github.com/veitlab/moove Download Data 1, ZIP file. [file eneuro-13-ENEURO.0023-26.2026-s001.zip › moove-main/docs/source/_static/images/image22.png]

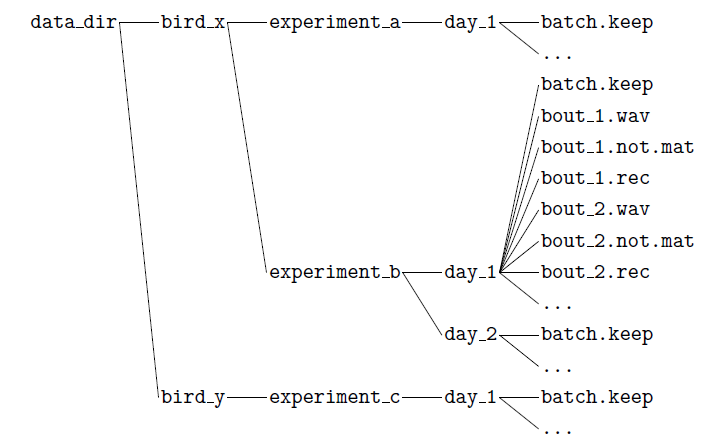

Supplement: Data 1 — The complete Python source code for MooveTAF (real-time recording and targeting) and MooveGUI (data preprocessing, labeling, and network training), packaged as a single ZIP archive. The latest version is available at: https://github.com/veitlab/moove Download Data 1, ZIP file. [file eneuro-13-ENEURO.0023-26.2026-s001.zip › moove-main/docs/source/_static/images/image23.png]

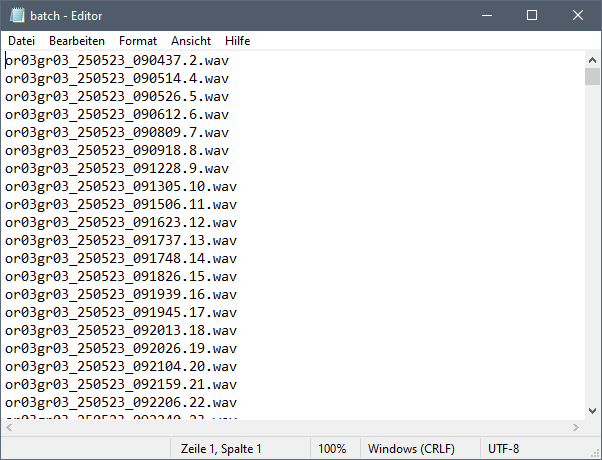

Supplement: Data 1 — The complete Python source code for MooveTAF (real-time recording and targeting) and MooveGUI (data preprocessing, labeling, and network training), packaged as a single ZIP archive. The latest version is available at: https://github.com/veitlab/moove Download Data 1, ZIP file. [file eneuro-13-ENEURO.0023-26.2026-s001.zip › moove-main/docs/source/_static/images/image24.png]

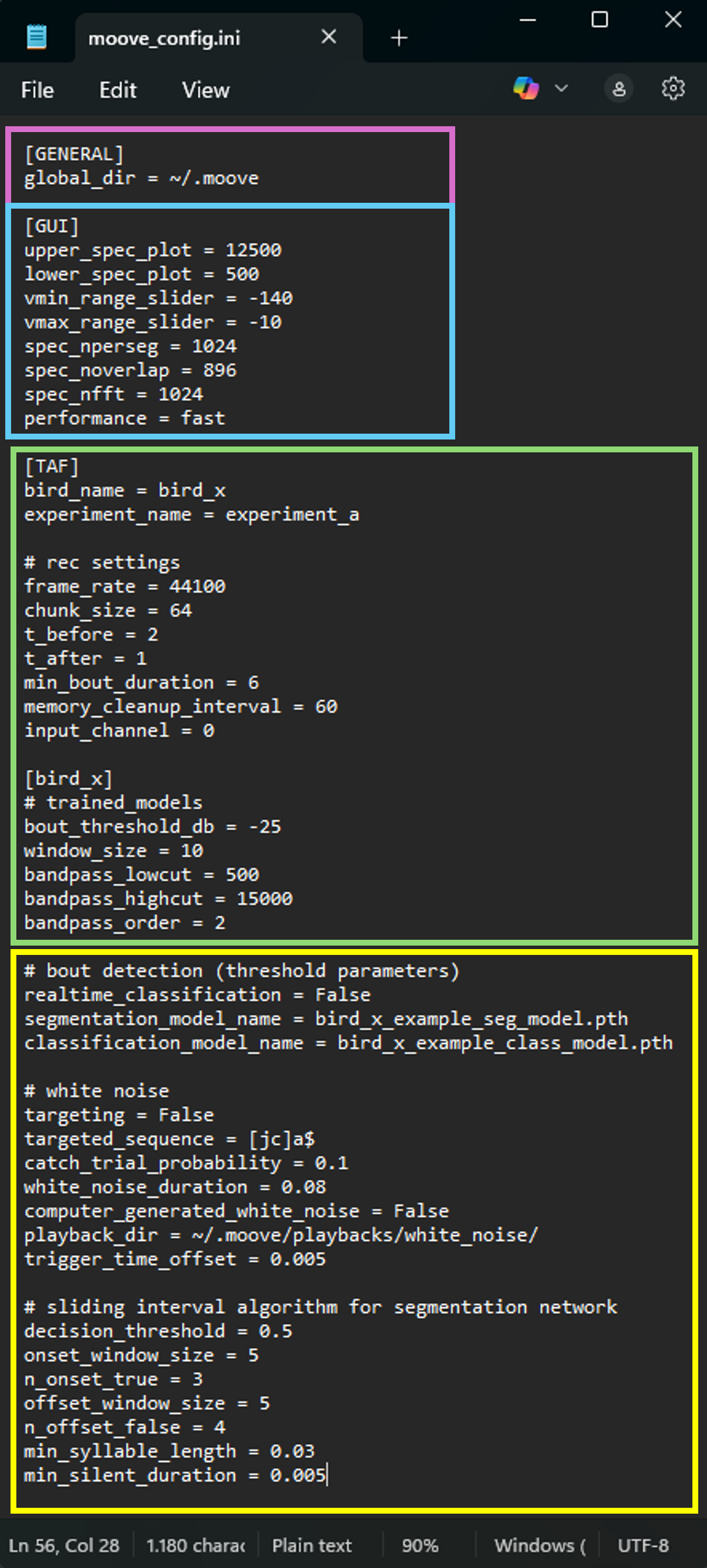

Supplement: Data 1 — The complete Python source code for MooveTAF (real-time recording and targeting) and MooveGUI (data preprocessing, labeling, and network training), packaged as a single ZIP archive. The latest version is available at: https://github.com/veitlab/moove Download Data 1, ZIP file. [file eneuro-13-ENEURO.0023-26.2026-s001.zip › moove-main/docs/source/_static/images/image25.png]

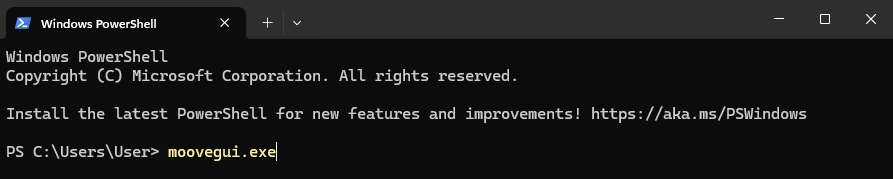

Supplement: Data 1 — The complete Python source code for MooveTAF (real-time recording and targeting) and MooveGUI (data preprocessing, labeling, and network training), packaged as a single ZIP archive. The latest version is available at: https://github.com/veitlab/moove Download Data 1, ZIP file. [file eneuro-13-ENEURO.0023-26.2026-s001.zip › moove-main/docs/source/_static/images/image26.png]

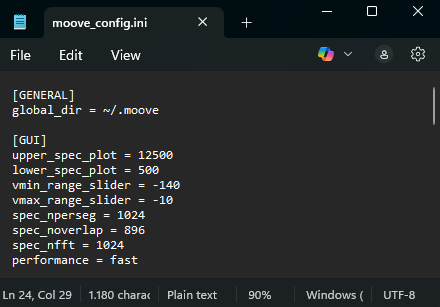

Supplement: Data 1 — The complete Python source code for MooveTAF (real-time recording and targeting) and MooveGUI (data preprocessing, labeling, and network training), packaged as a single ZIP archive. The latest version is available at: https://github.com/veitlab/moove Download Data 1, ZIP file. [file eneuro-13-ENEURO.0023-26.2026-s001.zip › moove-main/docs/source/_static/images/image27.png]

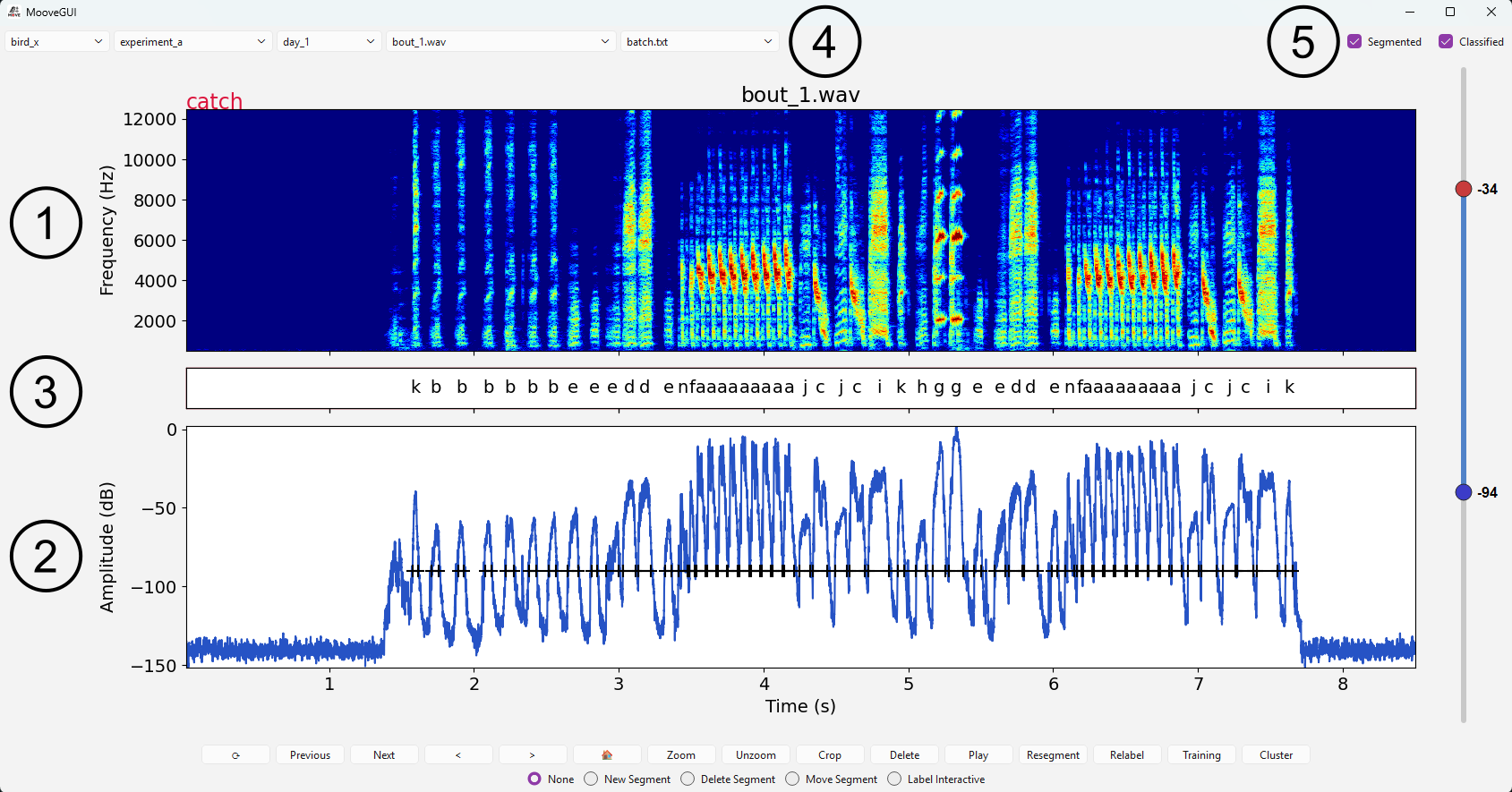

Supplement: Data 1 — The complete Python source code for MooveTAF (real-time recording and targeting) and MooveGUI (data preprocessing, labeling, and network training), packaged as a single ZIP archive. The latest version is available at: https://github.com/veitlab/moove Download Data 1, ZIP file. [file eneuro-13-ENEURO.0023-26.2026-s001.zip › moove-main/docs/source/_static/images/image28.png]

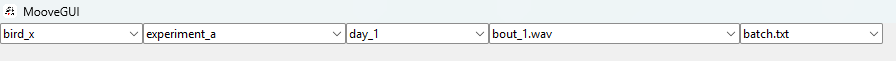

Supplement: Data 1 — The complete Python source code for MooveTAF (real-time recording and targeting) and MooveGUI (data preprocessing, labeling, and network training), packaged as a single ZIP archive. The latest version is available at: https://github.com/veitlab/moove Download Data 1, ZIP file. [file eneuro-13-ENEURO.0023-26.2026-s001.zip › moove-main/docs/source/_static/images/image29.png]

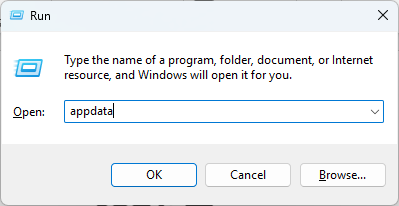

Supplement: Data 1 — The complete Python source code for MooveTAF (real-time recording and targeting) and MooveGUI (data preprocessing, labeling, and network training), packaged as a single ZIP archive. The latest version is available at: https://github.com/veitlab/moove Download Data 1, ZIP file. [file eneuro-13-ENEURO.0023-26.2026-s001.zip › moove-main/docs/source/_static/images/image3.png]

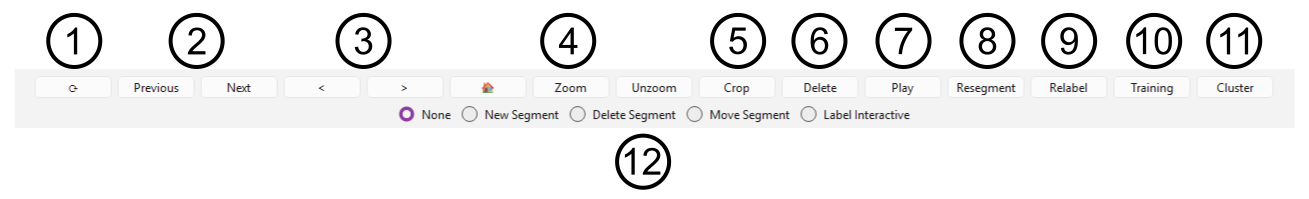

Supplement: Data 1 — The complete Python source code for MooveTAF (real-time recording and targeting) and MooveGUI (data preprocessing, labeling, and network training), packaged as a single ZIP archive. The latest version is available at: https://github.com/veitlab/moove Download Data 1, ZIP file. [file eneuro-13-ENEURO.0023-26.2026-s001.zip › moove-main/docs/source/_static/images/image30.png]

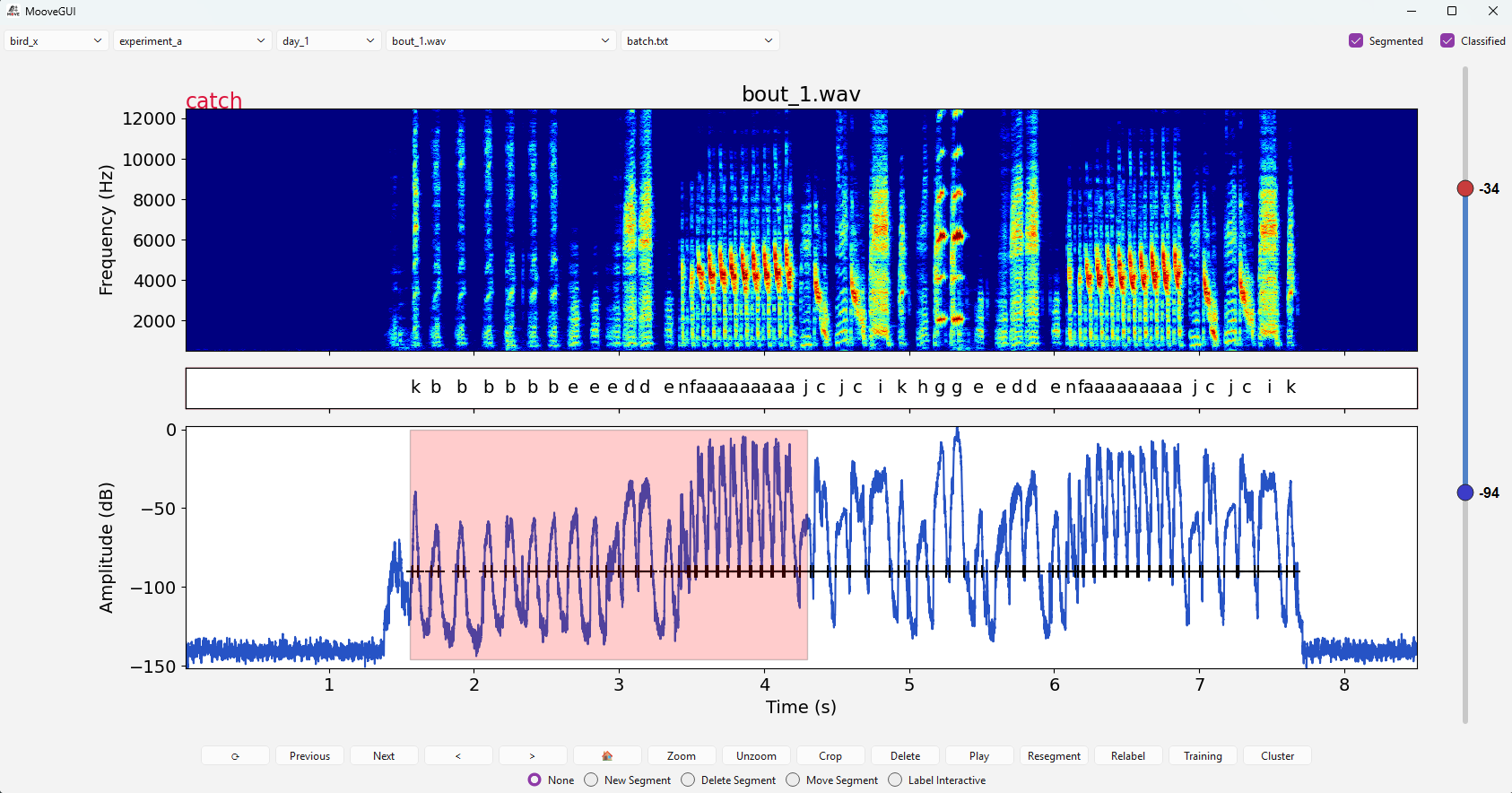

Supplement: Data 1 — The complete Python source code for MooveTAF (real-time recording and targeting) and MooveGUI (data preprocessing, labeling, and network training), packaged as a single ZIP archive. The latest version is available at: https://github.com/veitlab/moove Download Data 1, ZIP file. [file eneuro-13-ENEURO.0023-26.2026-s001.zip › moove-main/docs/source/_static/images/image31.png]

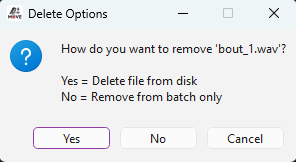

Supplement: Data 1 — The complete Python source code for MooveTAF (real-time recording and targeting) and MooveGUI (data preprocessing, labeling, and network training), packaged as a single ZIP archive. The latest version is available at: https://github.com/veitlab/moove Download Data 1, ZIP file. [file eneuro-13-ENEURO.0023-26.2026-s001.zip › moove-main/docs/source/_static/images/image32.png]

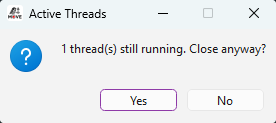

Supplement: Data 1 — The complete Python source code for MooveTAF (real-time recording and targeting) and MooveGUI (data preprocessing, labeling, and network training), packaged as a single ZIP archive. The latest version is available at: https://github.com/veitlab/moove Download Data 1, ZIP file. [file eneuro-13-ENEURO.0023-26.2026-s001.zip › moove-main/docs/source/_static/images/image33.png]

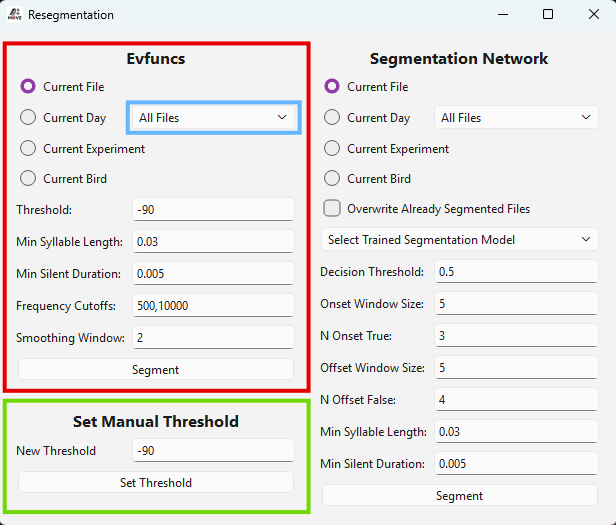

Supplement: Data 1 — The complete Python source code for MooveTAF (real-time recording and targeting) and MooveGUI (data preprocessing, labeling, and network training), packaged as a single ZIP archive. The latest version is available at: https://github.com/veitlab/moove Download Data 1, ZIP file. [file eneuro-13-ENEURO.0023-26.2026-s001.zip › moove-main/docs/source/_static/images/image34.png]

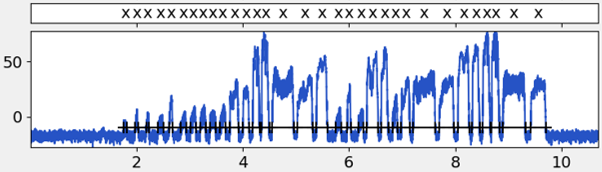

Supplement: Data 1 — The complete Python source code for MooveTAF (real-time recording and targeting) and MooveGUI (data preprocessing, labeling, and network training), packaged as a single ZIP archive. The latest version is available at: https://github.com/veitlab/moove Download Data 1, ZIP file. [file eneuro-13-ENEURO.0023-26.2026-s001.zip › moove-main/docs/source/_static/images/image35.png]

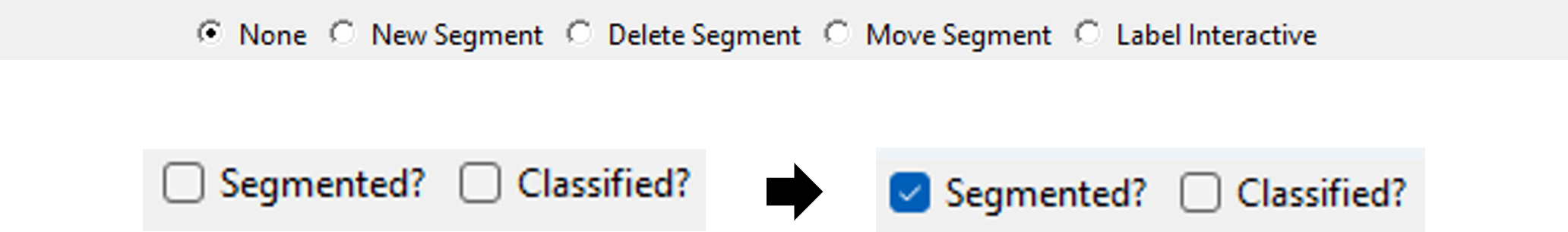

Supplement: Data 1 — The complete Python source code for MooveTAF (real-time recording and targeting) and MooveGUI (data preprocessing, labeling, and network training), packaged as a single ZIP archive. The latest version is available at: https://github.com/veitlab/moove Download Data 1, ZIP file. [file eneuro-13-ENEURO.0023-26.2026-s001.zip › moove-main/docs/source/_static/images/image36.png]

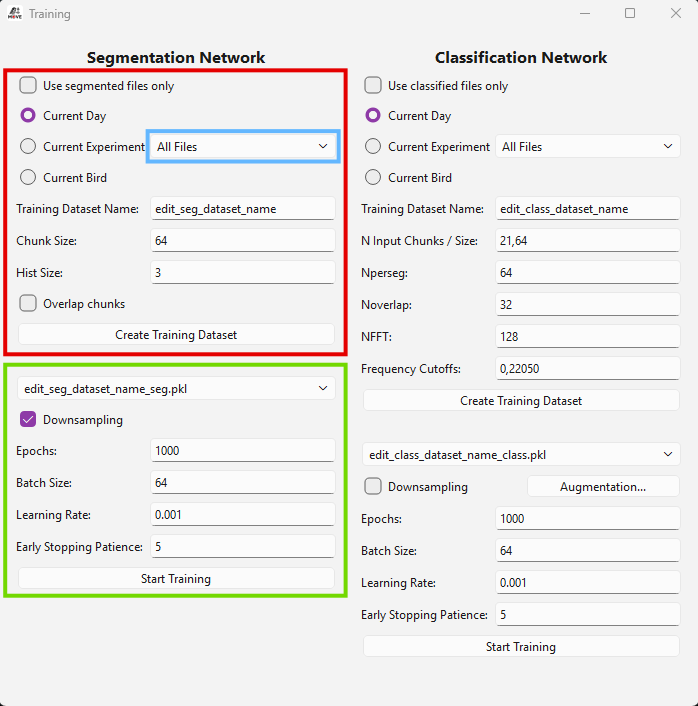

Supplement: Data 1 — The complete Python source code for MooveTAF (real-time recording and targeting) and MooveGUI (data preprocessing, labeling, and network training), packaged as a single ZIP archive. The latest version is available at: https://github.com/veitlab/moove Download Data 1, ZIP file. [file eneuro-13-ENEURO.0023-26.2026-s001.zip › moove-main/docs/source/_static/images/image37.png]

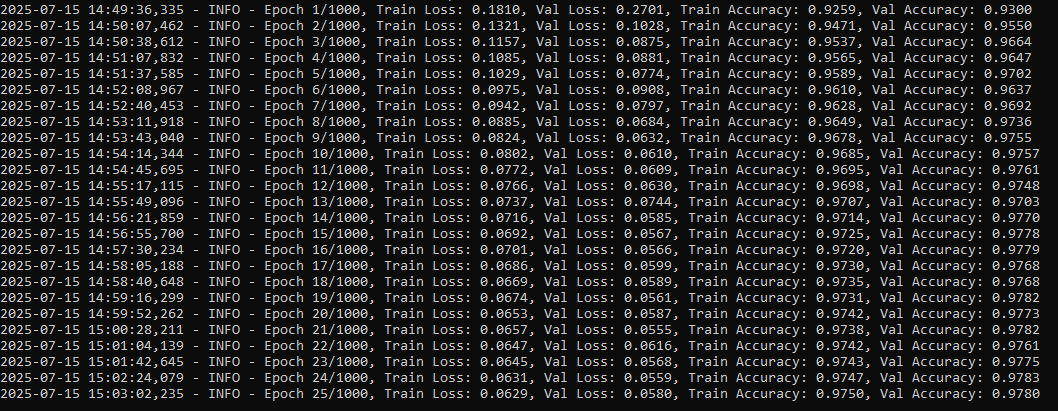

Supplement: Data 1 — The complete Python source code for MooveTAF (real-time recording and targeting) and MooveGUI (data preprocessing, labeling, and network training), packaged as a single ZIP archive. The latest version is available at: https://github.com/veitlab/moove Download Data 1, ZIP file. [file eneuro-13-ENEURO.0023-26.2026-s001.zip › moove-main/docs/source/_static/images/image38.png]

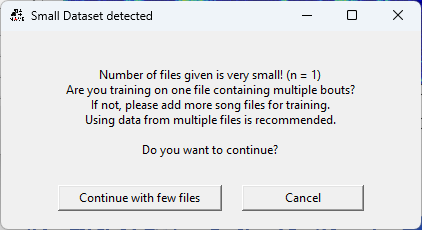

Supplement: Data 1 — The complete Python source code for MooveTAF (real-time recording and targeting) and MooveGUI (data preprocessing, labeling, and network training), packaged as a single ZIP archive. The latest version is available at: https://github.com/veitlab/moove Download Data 1, ZIP file. [file eneuro-13-ENEURO.0023-26.2026-s001.zip › moove-main/docs/source/_static/images/image39.png]

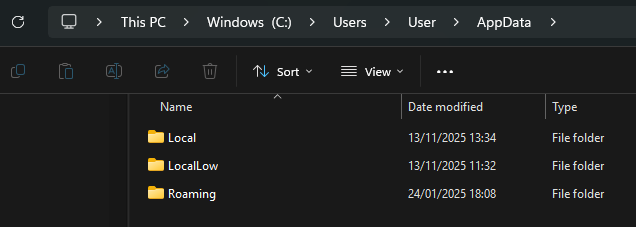

Supplement: Data 1 — The complete Python source code for MooveTAF (real-time recording and targeting) and MooveGUI (data preprocessing, labeling, and network training), packaged as a single ZIP archive. The latest version is available at: https://github.com/veitlab/moove Download Data 1, ZIP file. [file eneuro-13-ENEURO.0023-26.2026-s001.zip › moove-main/docs/source/_static/images/image4.png]

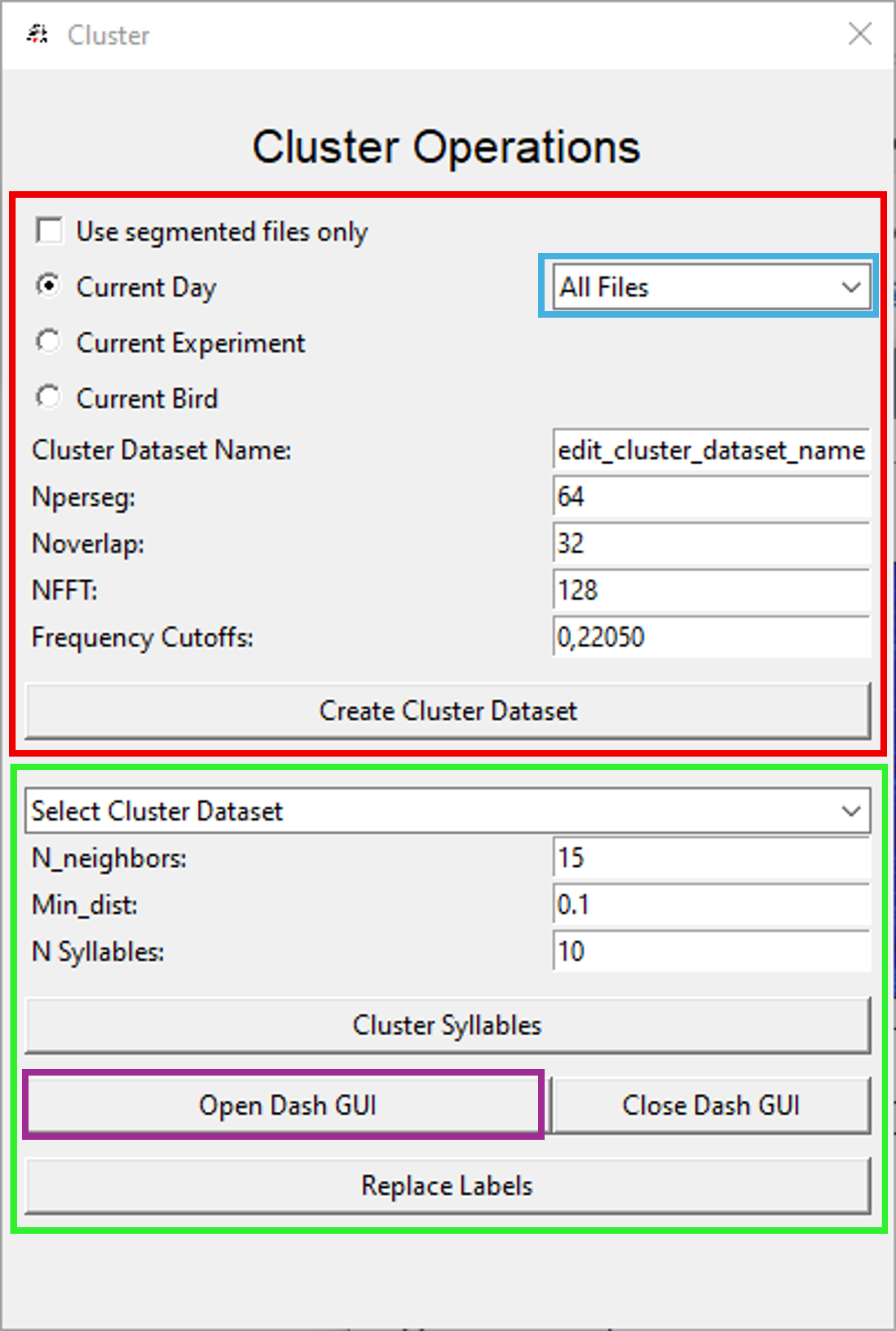

Supplement: Data 1 — The complete Python source code for MooveTAF (real-time recording and targeting) and MooveGUI (data preprocessing, labeling, and network training), packaged as a single ZIP archive. The latest version is available at: https://github.com/veitlab/moove Download Data 1, ZIP file. [file eneuro-13-ENEURO.0023-26.2026-s001.zip › moove-main/docs/source/_static/images/image40.png]

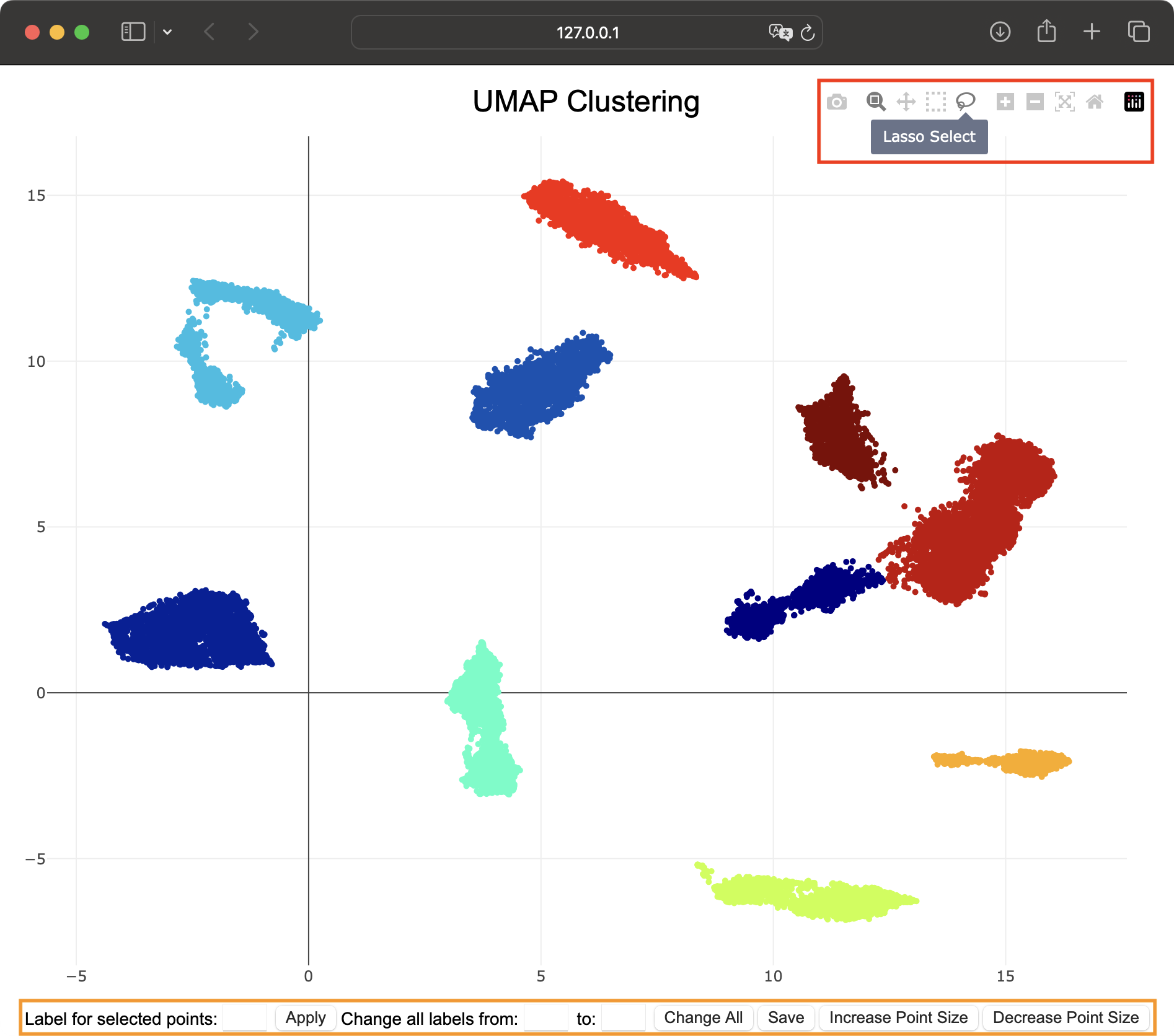

Supplement: Data 1 — The complete Python source code for MooveTAF (real-time recording and targeting) and MooveGUI (data preprocessing, labeling, and network training), packaged as a single ZIP archive. The latest version is available at: https://github.com/veitlab/moove Download Data 1, ZIP file. [file eneuro-13-ENEURO.0023-26.2026-s001.zip › moove-main/docs/source/_static/images/image41.png]

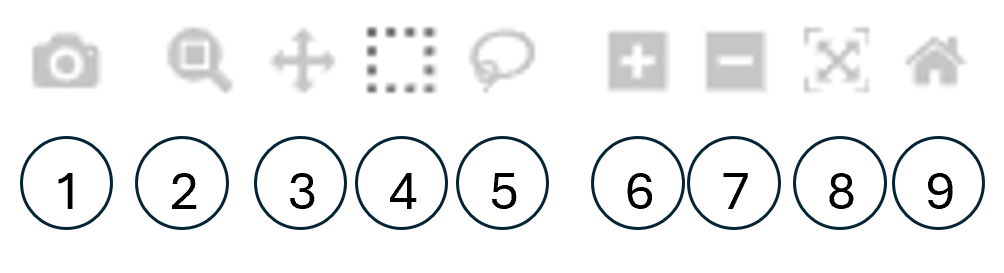

Supplement: Data 1 — The complete Python source code for MooveTAF (real-time recording and targeting) and MooveGUI (data preprocessing, labeling, and network training), packaged as a single ZIP archive. The latest version is available at: https://github.com/veitlab/moove Download Data 1, ZIP file. [file eneuro-13-ENEURO.0023-26.2026-s001.zip › moove-main/docs/source/_static/images/image42.png]

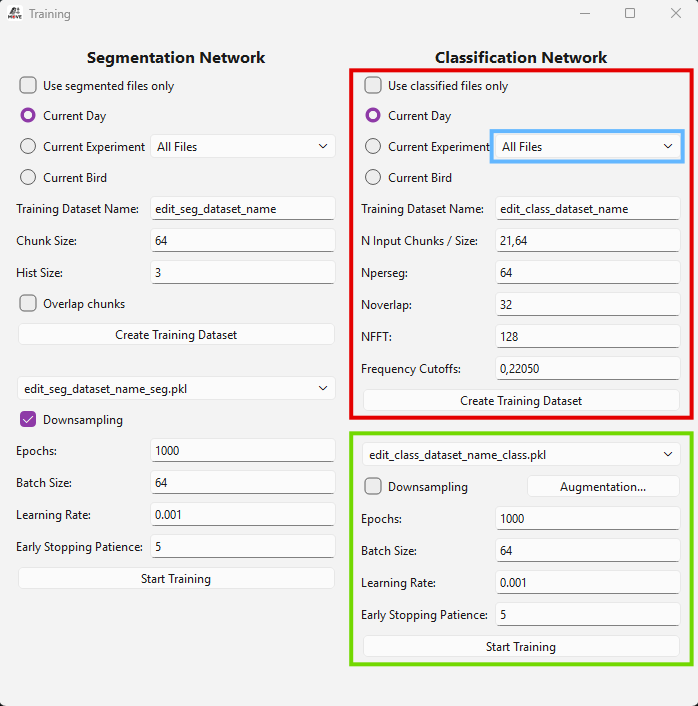

Supplement: Data 1 — The complete Python source code for MooveTAF (real-time recording and targeting) and MooveGUI (data preprocessing, labeling, and network training), packaged as a single ZIP archive. The latest version is available at: https://github.com/veitlab/moove Download Data 1, ZIP file. [file eneuro-13-ENEURO.0023-26.2026-s001.zip › moove-main/docs/source/_static/images/image43.png]

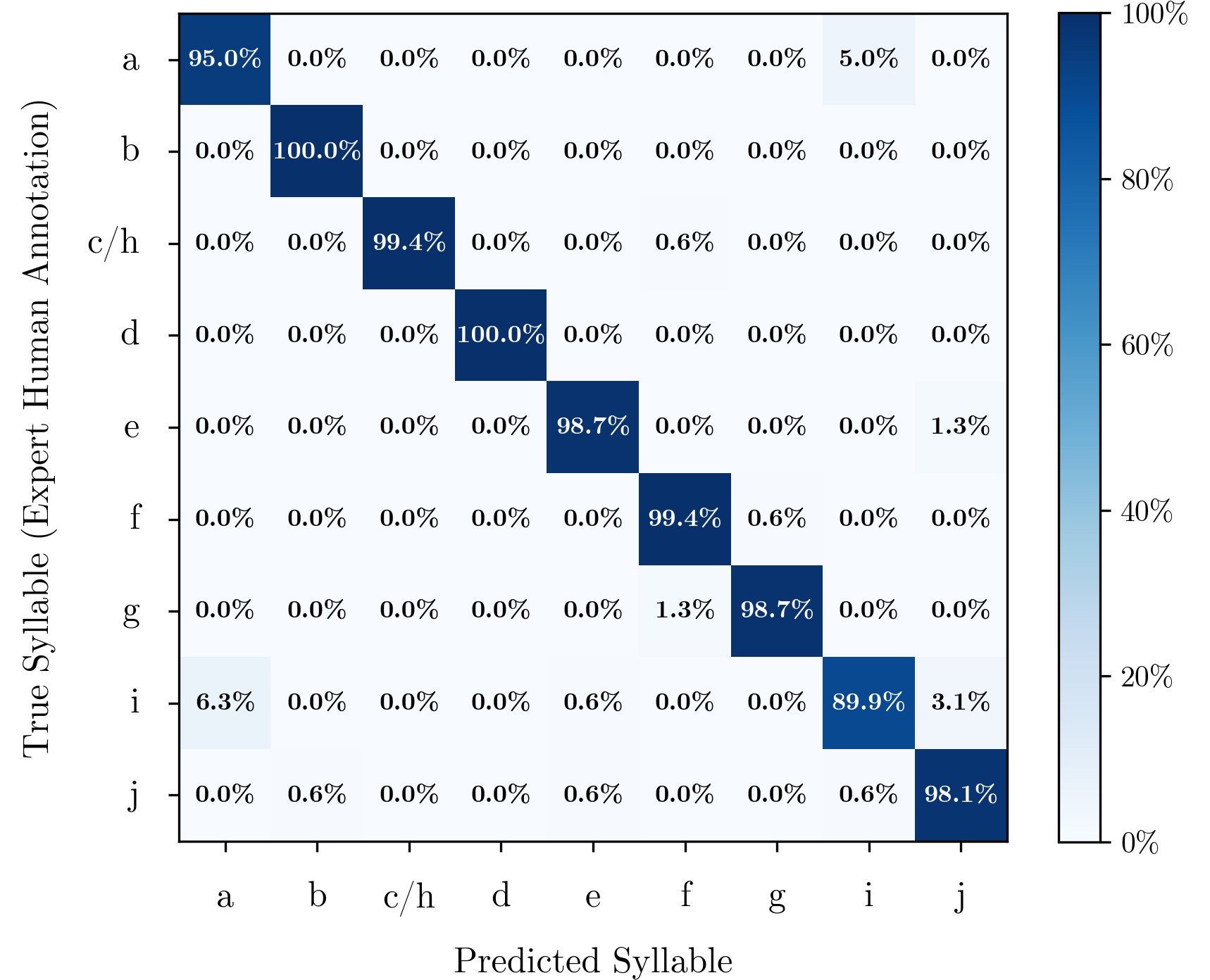

Supplement: Data 1 — The complete Python source code for MooveTAF (real-time recording and targeting) and MooveGUI (data preprocessing, labeling, and network training), packaged as a single ZIP archive. The latest version is available at: https://github.com/veitlab/moove Download Data 1, ZIP file. [file eneuro-13-ENEURO.0023-26.2026-s001.zip › moove-main/docs/source/_static/images/image44.jpg]

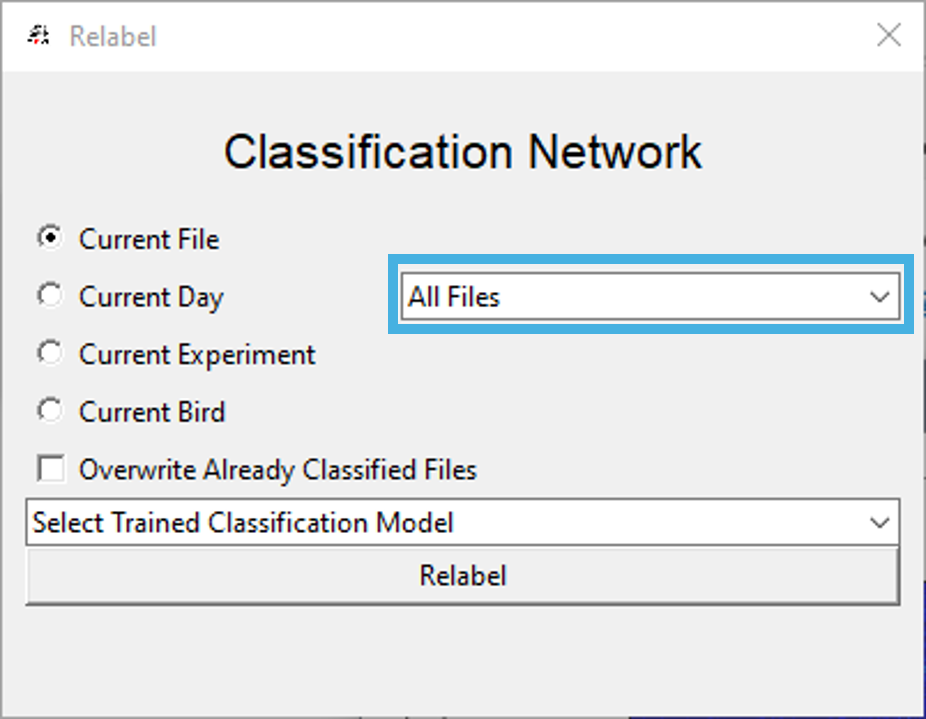

Supplement: Data 1 — The complete Python source code for MooveTAF (real-time recording and targeting) and MooveGUI (data preprocessing, labeling, and network training), packaged as a single ZIP archive. The latest version is available at: https://github.com/veitlab/moove Download Data 1, ZIP file. [file eneuro-13-ENEURO.0023-26.2026-s001.zip › moove-main/docs/source/_static/images/image45.png]

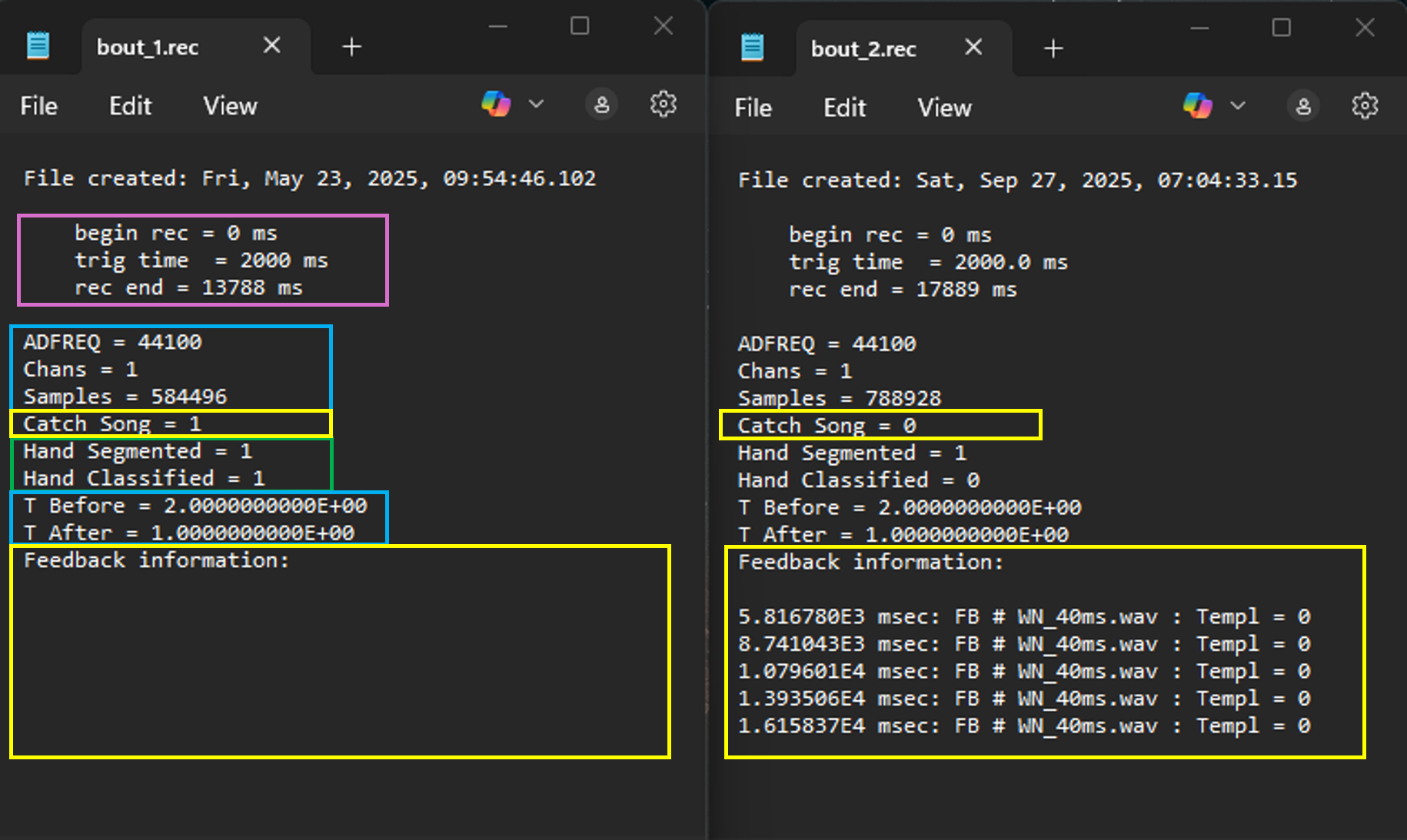

Supplement: Data 1 — The complete Python source code for MooveTAF (real-time recording and targeting) and MooveGUI (data preprocessing, labeling, and network training), packaged as a single ZIP archive. The latest version is available at: https://github.com/veitlab/moove Download Data 1, ZIP file. [file eneuro-13-ENEURO.0023-26.2026-s001.zip › moove-main/docs/source/_static/images/image46.png]

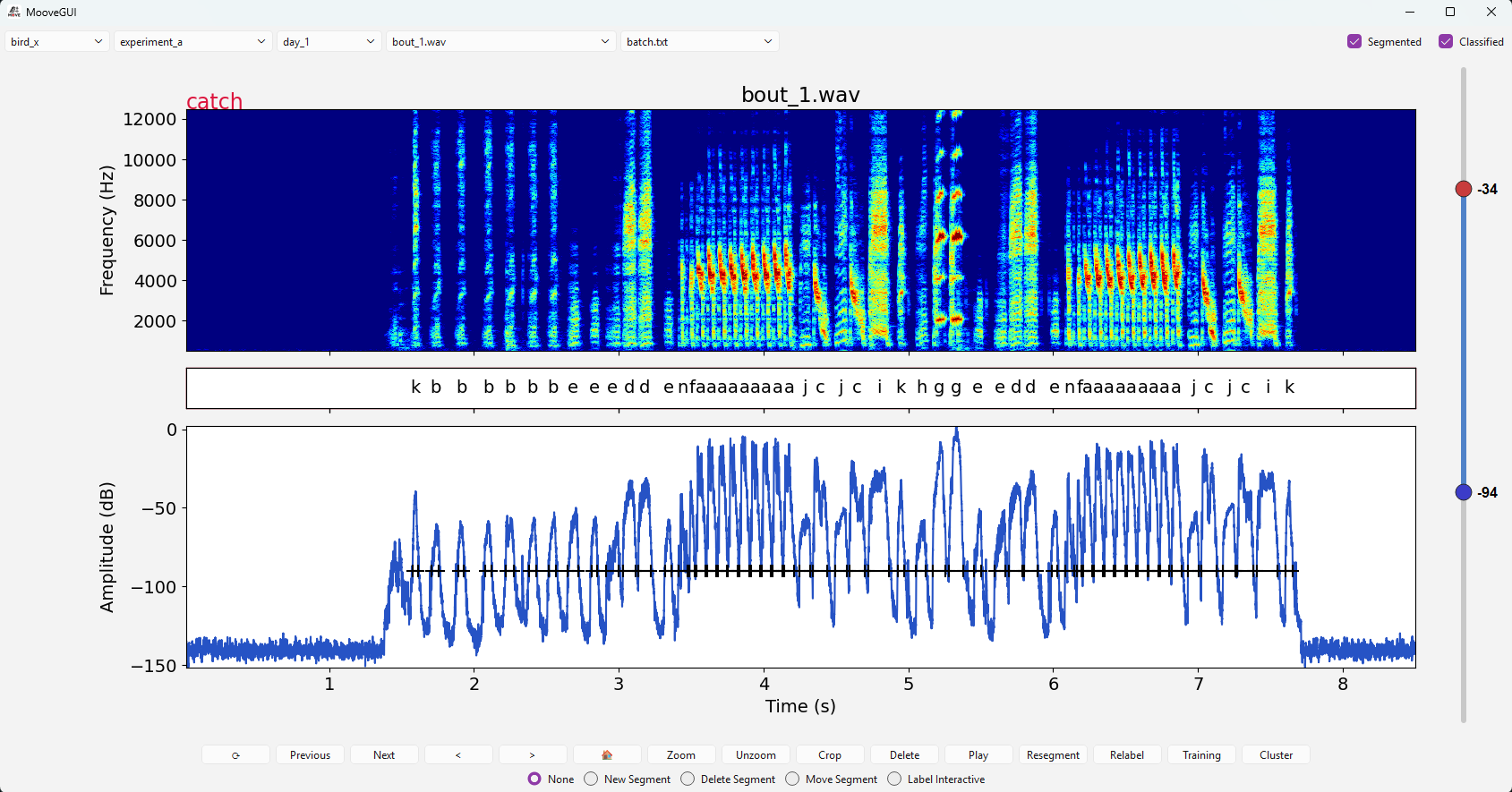

Supplement: Data 1 — The complete Python source code for MooveTAF (real-time recording and targeting) and MooveGUI (data preprocessing, labeling, and network training), packaged as a single ZIP archive. The latest version is available at: https://github.com/veitlab/moove Download Data 1, ZIP file. [file eneuro-13-ENEURO.0023-26.2026-s001.zip › moove-main/docs/source/_static/images/image47.png]

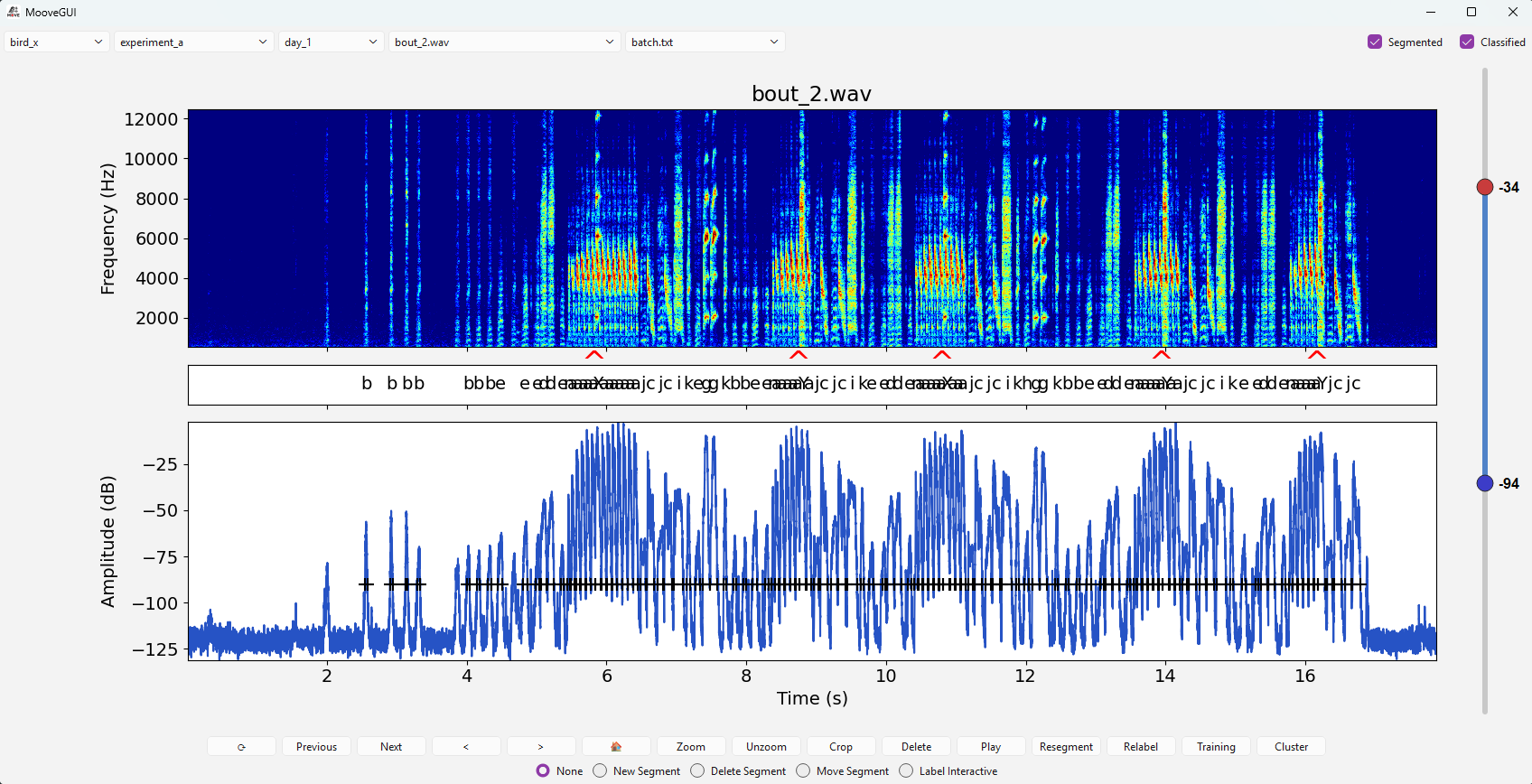

Supplement: Data 1 — The complete Python source code for MooveTAF (real-time recording and targeting) and MooveGUI (data preprocessing, labeling, and network training), packaged as a single ZIP archive. The latest version is available at: https://github.com/veitlab/moove Download Data 1, ZIP file. [file eneuro-13-ENEURO.0023-26.2026-s001.zip › moove-main/docs/source/_static/images/image48.png]

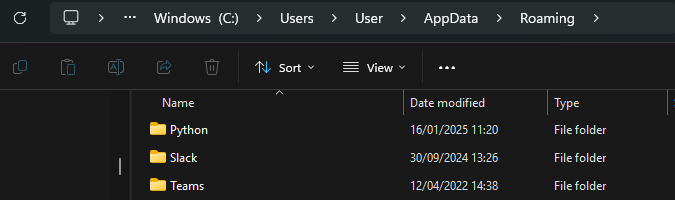

Supplement: Data 1 — The complete Python source code for MooveTAF (real-time recording and targeting) and MooveGUI (data preprocessing, labeling, and network training), packaged as a single ZIP archive. The latest version is available at: https://github.com/veitlab/moove Download Data 1, ZIP file. [file eneuro-13-ENEURO.0023-26.2026-s001.zip › moove-main/docs/source/_static/images/image5.png]

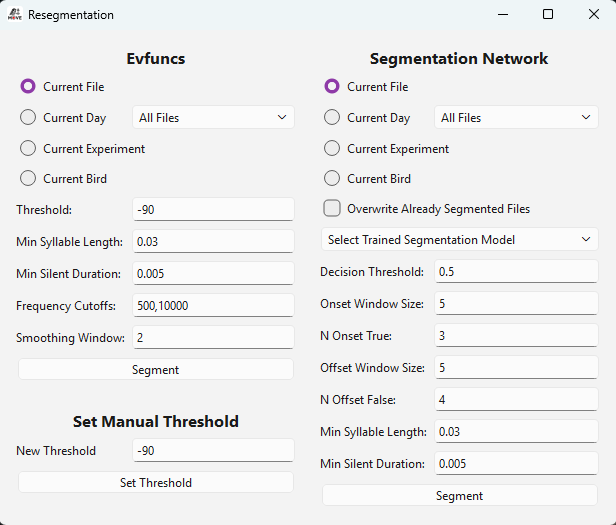

Supplement: Data 1 — The complete Python source code for MooveTAF (real-time recording and targeting) and MooveGUI (data preprocessing, labeling, and network training), packaged as a single ZIP archive. The latest version is available at: https://github.com/veitlab/moove Download Data 1, ZIP file. [file eneuro-13-ENEURO.0023-26.2026-s001.zip › moove-main/docs/source/_static/images/image53.png]

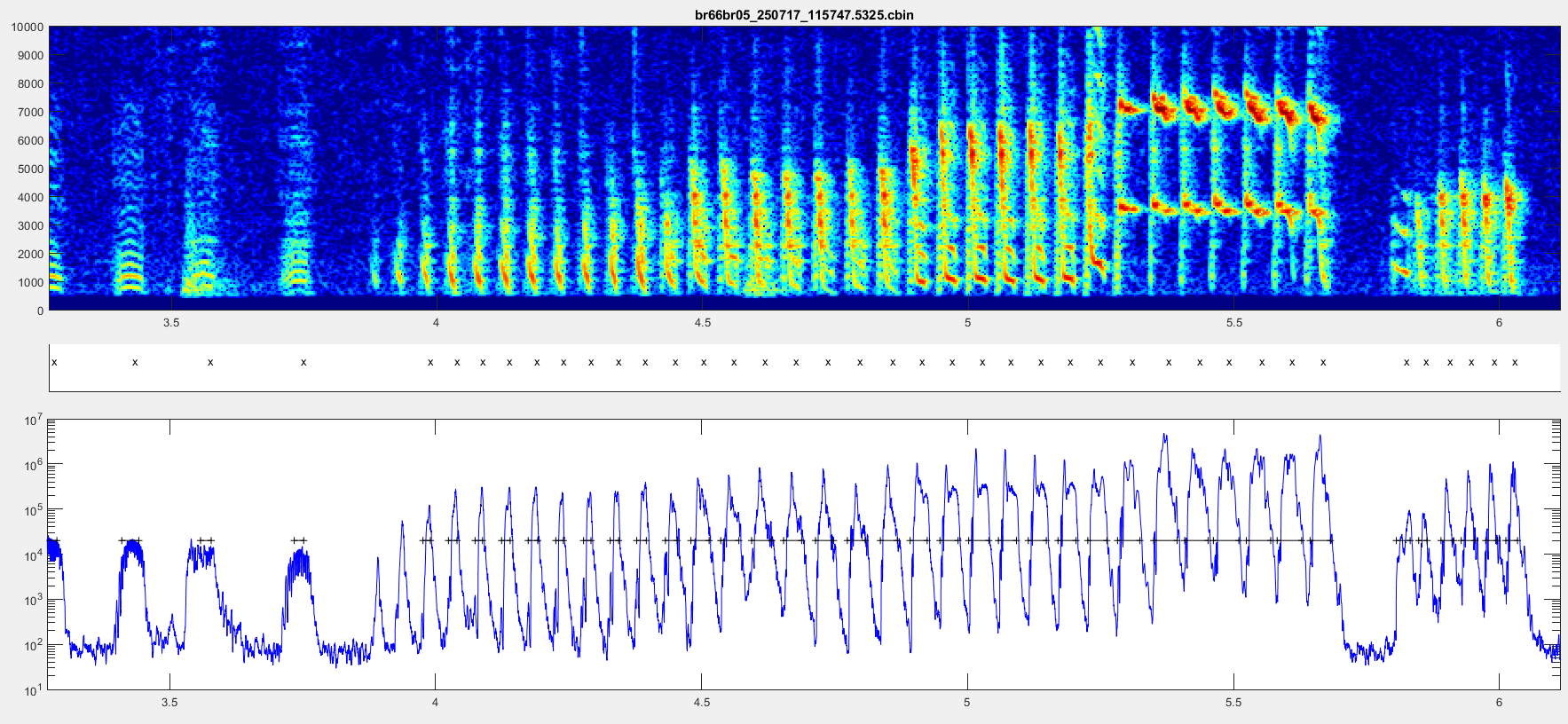

Supplement: Data 1 — The complete Python source code for MooveTAF (real-time recording and targeting) and MooveGUI (data preprocessing, labeling, and network training), packaged as a single ZIP archive. The latest version is available at: https://github.com/veitlab/moove Download Data 1, ZIP file. [file eneuro-13-ENEURO.0023-26.2026-s001.zip › moove-main/docs/source/_static/images/image54.png]

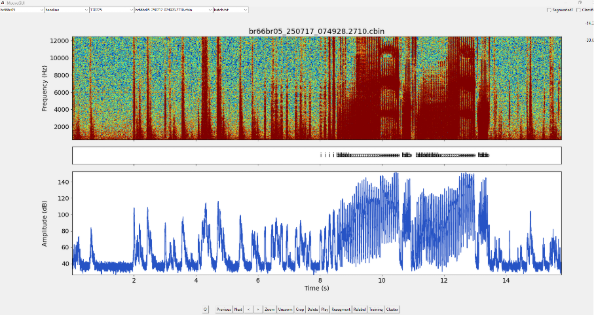

Supplement: Data 1 — The complete Python source code for MooveTAF (real-time recording and targeting) and MooveGUI (data preprocessing, labeling, and network training), packaged as a single ZIP archive. The latest version is available at: https://github.com/veitlab/moove Download Data 1, ZIP file. [file eneuro-13-ENEURO.0023-26.2026-s001.zip › moove-main/docs/source/_static/images/image55.png]

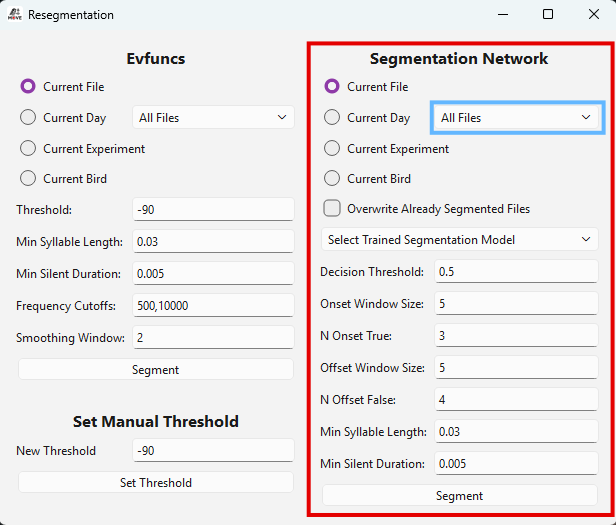

Supplement: Data 1 — The complete Python source code for MooveTAF (real-time recording and targeting) and MooveGUI (data preprocessing, labeling, and network training), packaged as a single ZIP archive. The latest version is available at: https://github.com/veitlab/moove Download Data 1, ZIP file. [file eneuro-13-ENEURO.0023-26.2026-s001.zip › moove-main/docs/source/_static/images/image56.png]

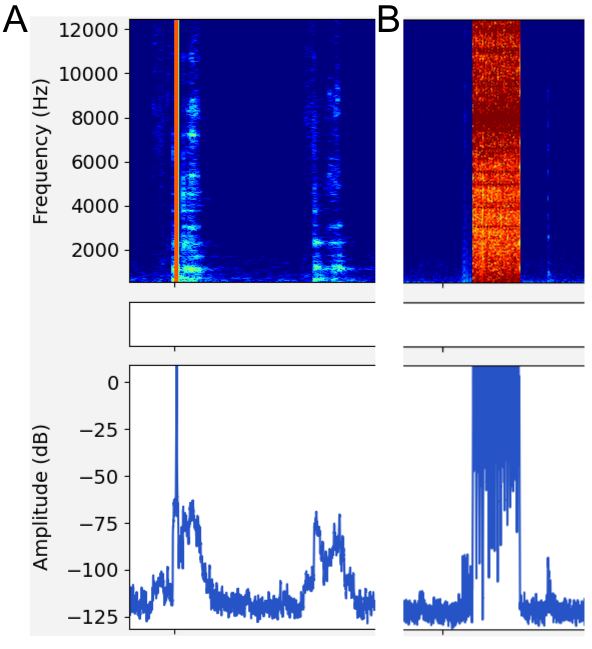

Supplement: Data 1 — The complete Python source code for MooveTAF (real-time recording and targeting) and MooveGUI (data preprocessing, labeling, and network training), packaged as a single ZIP archive. The latest version is available at: https://github.com/veitlab/moove Download Data 1, ZIP file. [file eneuro-13-ENEURO.0023-26.2026-s001.zip › moove-main/docs/source/_static/images/image57.png]

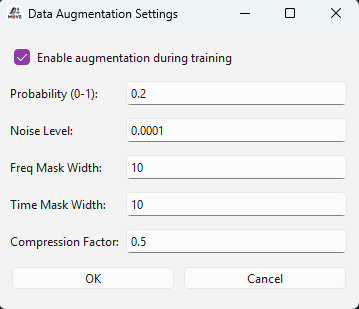

Supplement: Data 1 — The complete Python source code for MooveTAF (real-time recording and targeting) and MooveGUI (data preprocessing, labeling, and network training), packaged as a single ZIP archive. The latest version is available at: https://github.com/veitlab/moove Download Data 1, ZIP file. [file eneuro-13-ENEURO.0023-26.2026-s001.zip › moove-main/docs/source/_static/images/image58.png]

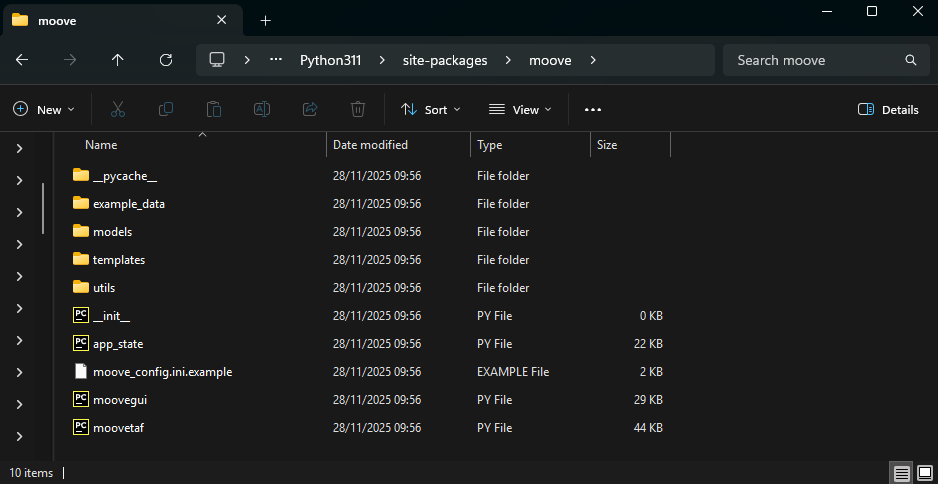

Supplement: Data 1 — The complete Python source code for MooveTAF (real-time recording and targeting) and MooveGUI (data preprocessing, labeling, and network training), packaged as a single ZIP archive. The latest version is available at: https://github.com/veitlab/moove Download Data 1, ZIP file. [file eneuro-13-ENEURO.0023-26.2026-s001.zip › moove-main/docs/source/_static/images/image6.png]

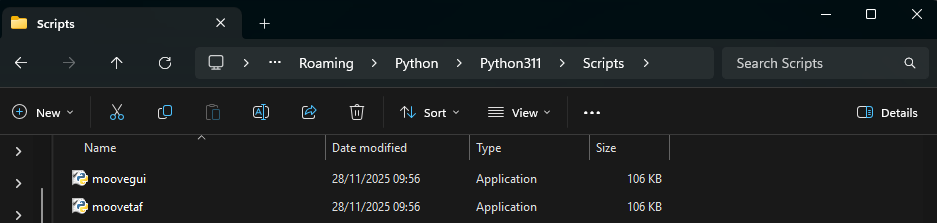

Supplement: Data 1 — The complete Python source code for MooveTAF (real-time recording and targeting) and MooveGUI (data preprocessing, labeling, and network training), packaged as a single ZIP archive. The latest version is available at: https://github.com/veitlab/moove Download Data 1, ZIP file. [file eneuro-13-ENEURO.0023-26.2026-s001.zip › moove-main/docs/source/_static/images/image7.png]

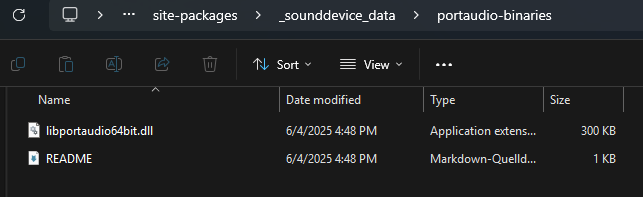

Supplement: Data 1 — The complete Python source code for MooveTAF (real-time recording and targeting) and MooveGUI (data preprocessing, labeling, and network training), packaged as a single ZIP archive. The latest version is available at: https://github.com/veitlab/moove Download Data 1, ZIP file. [file eneuro-13-ENEURO.0023-26.2026-s001.zip › moove-main/docs/source/_static/images/image8.png]

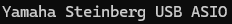

Supplement: Data 1 — The complete Python source code for MooveTAF (real-time recording and targeting) and MooveGUI (data preprocessing, labeling, and network training), packaged as a single ZIP archive. The latest version is available at: https://github.com/veitlab/moove Download Data 1, ZIP file. [file eneuro-13-ENEURO.0023-26.2026-s001.zip › moove-main/docs/source/_static/images/image9.png]
